# Impact of Dietary Polyphenols on Carbohydrate Metabolism

**DOI:** 10.3390/ijms11041365

**Published:** 2010-03-31

**Authors:** Kati Hanhineva, Riitta Törrönen, Isabel Bondia-Pons, Jenna Pekkinen, Marjukka Kolehmainen, Hannu Mykkänen, Kaisa Poutanen

**Affiliations:** 1 Department of Clinical Nutrition and Food and Health Research Centre, University of Eastern Finland, P.O. Box 1627, 70210 Kuopio, Finland; E-Mails: riitta.torronen@uef.fi (R.T.); Isabel.BondiaPons@uef.fi (I.B.-P.); jenna.pekkinen@uef.fi (J.P.); marjukka.kolehmainen@uef.fi (M.K.); hannu.mykkanen@uef.fi (H.M.); kaisa.poutanen@vtt.fi (K.P.); 2 VTT Technical Research Centre of Finland, P.O. Box 1000, FI-02044 VTT, Finland

**Keywords:** diet, phytochemical, polyphenols, phenolic compounds, glucose metabolism, insulin sensitivity, glycemic response

## Abstract

Polyphenols, including flavonoids, phenolic acids, proanthocyanidins and resveratrol, are a large and heterogeneous group of phytochemicals in plant-based foods, such as tea, coffee, wine, cocoa, cereal grains, soy, fruits and berries. Growing evidence indicates that various dietary polyphenols may influence carbohydrate metabolism at many levels. In animal models and a limited number of human studies carried out so far, polyphenols and foods or beverages rich in polyphenols have attenuated postprandial glycemic responses and fasting hyperglycemia, and improved acute insulin secretion and insulin sensitivity. The possible mechanisms include inhibition of carbohydrate digestion and glucose absorption in the intestine, stimulation of insulin secretion from the pancreatic β–cells, modulation of glucose release from the liver, activation of insulin receptors and glucose uptake in the insulin-sensitive tissues, and modulation of intracellular signalling pathways and gene expression. The positive effects of polyphenols on glucose homeostasis observed in a large number of *in vitro* and animal models are supported by epidemiological evidence on polyphenol-rich diets. To confirm the implications of polyphenol consumption for prevention of insulin resistance, metabolic syndrome and eventually type 2 diabetes, human trials with well-defined diets, controlled study designs and clinically relevant end-points together with holistic approaches e.g., systems biology profiling technologies are needed.

## 1. Introduction

Polyphenols are a large and heterogeneous group of phytochemicals of plant-based foods, such as tea, coffee, wine, cereal grains, vegetables, legumes, fruits and berries. The structural diversity of polyphenols extends from simple one-phenol hydroxybenzoic and hydroxycinnamic acids to large polymeric macromolecules like proanthocyanidins and ellagitannins. An essential group of phenolic compounds are flavonoids encompassing structural classes like flavonols, flavones, flavanols, flavanones, anthocyanidins and isoflavones. The estimated intake of dietary polyphenols is approximately 1 g/day [1]. Consumption of plant foods is associated with lowered risk of major chronic diseases including diabetes, cardiovascular diseases and cancer [2–5]. *In vitro* and *in vivo* studies on polyphenols show that polyphenols possess anti-inflammatory, antioxidative, chemopreventive and neuroprotective activities, suggesting that they could contribute to the health-protective properties of plant foods. Growing evidence indicates that dietary polyphenols also influence glucose and lipid metabolism

The majority of dietary polyphenols are metabolised by colonic microbiota before absorption, only smaller amount being absorbed directly from upper gastrointestinal tract [6]. Gut bacteria modulate polyphenols by various mechanisms including hydrolysis, ring-cleavage, reduction, decarboxylation and demethylation. The microbial metabolism is a pre-requisite for absorption, and it also modulates the biological activity of the compounds. The systemic effects of dietary polyphenols depend largely on the synergistic action that polyphenols may exert after entering circulation, and are affected by other constituents present in the diet as well as endogenous factors [7,8].

Starch and sucrose are the most important dietary carbohydrates. Their digestion, absorption and metabolism may be influenced by dietary polyphenols and their metabolites. Most dietary carbohydrate is digested in the upper gastrointestinal tract to monosaccharides which are then absorbed to the circulation. The elevated glucose concentration in blood promotes secretion of insulin from the β-cells of the islets of Langerhans in the pancreas, and insulin mediates the uptake of glucose in peripheral tissues including muscle, adipose tissue and kidney, promotes storage of glucose in liver as glycogen, and inhibits lipolysis in adipose tissue. Another essential hormone in maintaining the glucose homeostasis is glucagon that is secreted from the pancreatic α-cells once the blood glucose level begins to fall below normal. Glucagon promotes liver glucose production by inducing glycogenolysis and gluconeogenesis to ensure adequate circulating glucose to fuel the body functions.

Maintenance of glucose homeostasis is of utmost importance to human physiology, being under strict hormonal control. Failure of this control can result in the metabolic syndrome, a multi-symptom disorder of energy homeostasis encompassing obesity, hyperglycemia, impaired glucose tolerance, hypertension and dyslipidemia [9]. The most characteristic abnormality in the metabolic syndrome is insulin resistance, which results from interactions between genetic and environmental factors, including diet and sedentary lifestyle [10,11]. Metabolic syndrome is the major predisposing factor to type 2 diabetes, where defects in both insulin action and insulin secretion are present, but their relative contribution varies individually. The disturbance of glucose metabolism is often related to the increase of fat mass, especially in the abdominal area and ectopically, to the tissues where fat is not stored in normal energy homeostasis [12]. This in turn results in inflammation and exacerbated oxidative stress at the whole body level, and malfunction in several organs including pancreas, liver, muscle and adipose tissue [13].

**Figure 1.**
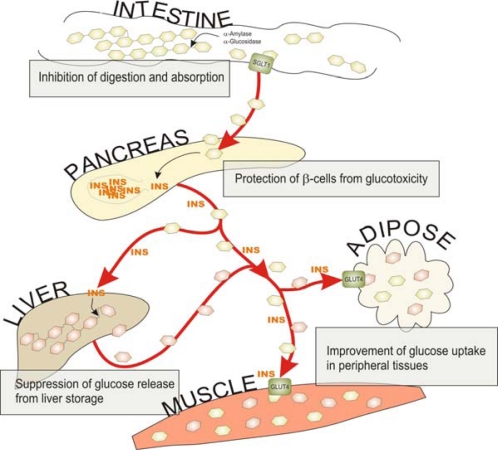
Potential sites of action of dietary polyphenols on carbohydrate metabolism and glucose homeostasis.

The prevalence of type 2 diabetes is rising exponentially, estimated to reach over 300 million cases by year 2030 [14]. Presently, the treatment of metabolic syndrome and prevention of type 2 diabetes involves lifestyle modifications like increased physical activity and weight control by reduced caloric intake [15,16]. Increasingly, the dietary recommendations for individuals at risk of type 2 diabetes emphasise the intake of plant food products, such as whole grains, berries, fruits and vegetables, all known to be excellent sources of dietary fibre, but also good sources of variable polyphenolic compounds. These compounds may influence glucose metabolism by several mechanisms, such as inhibition of carbohydrate digestion and glucose absorption in the intestine, stimulation of insulin secretion from the pancreatic β–cells, modulation of glucose release from liver, activation of insulin receptors and glucose uptake in the insulin-sensitive tissues, and modulation of hepatic glucose output (Figure 1).

Dietary polyphenols are found in distinctive combinations of metabolites from different chemical classes. The biochemical properties and resulting health-beneficial bioactivities in different plant groups or even different species are thus discrete, having different impact on different health conditions [3]. In terms of metabolic syndrome and type 2 diabetes, the up-to-date most extensively studied plants and metabolites include soy, that is one of the few edible plants having high concentrations of isoflavonoids [17]; tea, mainly for condensed tannins, in particular epigallocatecin gallate [18]; coffee, for phenolic acid content [19]; grape especially for the presence of resveratrol [20]; apple for rich flavonoid content [21] and several herbs often possessing highly distinct phytochemical profiles, e.g., high content of terpenoids [22]. Also different berry species like cranberry, strawberry and blueberry have been addressed to possess capacity to protect from diabetes, and the studies have most often focused on the anthocyanin metabolite class [23]. Similarly, the whole grain products are intensively studied not only for the high fibre content but also for the rich phenolic compound repertoire that may have beneficial effect on glucose homeostasis [24]. Whilst the results from dietary human interventions are still scarce, there is a wealthy of data published with different diabetic animal models. The most common ones are rat and mice models with diet-induced diabetes, thereby resembling the type 2 diabetes in humans, and the models with destruction of pancreas by allozan or streptotozin treatment resulting in insulin deficiency. Various *in vitro* studies have been performed by different cell lines of adipose, hepatic, pancreatic and myotube origin.

This review will demonstrate the potential of dietary phenolic phytochemicals in maintenance of glucose and energy homeostasis and in suppression of metabolic syndrome and type 2 diabetes as evidenced by rapidly expanding literature. However, the antioxidant role of these compounds in metabolic syndrome, extensively reviewed recently [25,26], is not discussed herein.

## 2. Influence of Polyphenols on Carbohydrate Digestion and Glucose Absorption in the Intestine

Food and beverages high in available carbohydrates such as starch or sucrose induce postprandial hyperglycemia, hyperinsulinemia and other hormonal and metabolic disturbances. The rapid absorption of glucose challenges the regulatory mechanisms of glucose homeostasis, and habitual consumption of high-glycemic diets may therefore increase the risk for obesity, type 2 diabetes and cardiovascular disease [27]. Carbohydrate digestion and glucose absorption are obvious targets for better glycemia control after high-carbohydrate meals. α-Amylase and α-glucosidase are the key enzymes responsible for digestion of dietary carbohydrates to glucose. The liberated glucose is absorbed across the intestinal enterocytes *via* specific transporters. Inhibition of the digestive enzymes or glucose transporters would reduce the rate of glucose release and absorption in the small intestine and consequently suppress postprandial hyperglycemia.

### 2.1. Carbohydrate Digestion

Starch is composed of amylose, which is a linear α-1,4-linked glucose polymer, and highly branched amylopectin consisting of linear α-1,4-linked glucose chains with α-1,6-linked branch chains. Salivary and pancreatic α-amylases catalyze the endo-hydrolysis of α-1,4-glucosidic linkages releasing mainly maltose, maltotriose and related α-1,6-oligomers. Further digestion takes place in the small intestinal brush border by α-glucosidases, which hydrolyze the terminal α-1,4-linked glucose residues as the final step in the digestion of dietary carbohydrates to release glucose. The α-glucosidase activities, first described as maltases, are associated with maltase-glucoamylase and sucrase-isomaltase [28–30]. In addition to α-1,4-glucosidic activity, sucrase-isomaltase displays specific activities against the α-1,2 linkages of sucrose and α-1,6 linkages of isomaltose.

A variety of polyphenols have been shown to inhibit α-amylase and α-glucosidase activities *in vitro* (Table 1). The inhibitory polyphenols include flavonoids (anthocyanins, catechins, flavanones, flavonols, flavones and isoflavones), phenolic acids and tannins (proanthocyanidins and ellagitannins). In addition, *in vitro* inhibitory activities have been reported for polyphenolic extracts of foods, including berries (strawberries, raspberries, blueberries and blackcurrants), vegetables (pumpkin, beans, maize and eggplant), colored grains such as black rice, green and black tea, and red wine (Table 2). In the studies, maltose, sucrose or *p*-nitrophenyl-α-d-glucopyranoside have been used as substrate for α-glucosidase activity.

### 2.2. Glucose Absorption

Intestinal absorption of glucose is mediated by active transport *via* the sodium-dependent glucose transporter SGLT1 and by facilitated sodium-independent transport *via* the glucose transporter GLUT2 [31,32]. On the luminal side of the intestinal brush border membrane, two Na^+^ ions bind to SGLT1 and produce a conformational change that permits glucose binding, followed by another conformational change to allow glucose and Na^+^ to enter the enterocyte. Glucose is released from the enterocyte *via* GLUT2, a high capacity facilitative transporter in the basolateral membrane, to enter the circulation.

The influence of polyphenols on glucose transporters has been studied *in vitro* by using intestinal brush border membrane vesicles or everted sacs and Caco-2 cells. Several flavonoids and phenolic acids have been shown to inhibit glucose transport (Table 2). The Na^+^-dependent SGLT1-mediated glucose transport was inhibited by chlorogenic, ferulic, caffeic and tannic acids [33], quercetin monoglucosides [34], tea catechins [35–37] and naringenin [38]. The glucose transport by GLUT2 was inhibited by quercetin, myricetin, apigenin and tea catechins [37,39].

### 2.3. Postprandial Glycemia

Effects of polyphenols, polyphenolic food fractions, and foods and beverages rich in polyphenols on postprandial blood glucose responses have been investigated in animal models and in human studies. Either glucose, maltose, sucrose, starch or various meals have been used as the carbohydrate challenge.

Animal studies. Diacylated anthocyanin as well as an anthocyanin extract from purple sweet potato reduced the blood glucose and insulin responses to maltose administration in rats [40]. The lack of effect after sucrose or glucose administration indicates that the anti-hyperglycemic effect was achieved by maltase inhibition, and not by inhibition of intestinal sucrase activity or glucose transport. Also a tea polyphenol, theaflavin 3-O-gallate, was effective in suppressing the postprandial glucose response to maltose [41].

A crude Acerola polyphenol fraction (containing anthocyanins) significantly reduced the plasma glucose level after administration of maltose or glucose in mice, suggesting inhibition of α-glucosidase and intestinal glucose transport [42]. A leaf extract of *Nerium indicum*, a plant used as a folk remedy for type 2 diabetes in Pakistan, was found to reduce the postprandial rise in blood glucose in maltose-or sucrose-loaded rats [43]. A similar response was obtained with chlorogenic acid, which was identified as the major α-glucosidase inhibitor in the leaf extract.

*Gingko biloba* extracts and their flavonoid fraction reduced the elevation of rat plasma glucose level after oral administration of starch, maltose, sucrose or glucose [44]. Also in diabetic rats, the flavonoid fraction attenuated the glucose response to sucrose and glucose administration. When diabetic rats were administered glucose with quercetin, hyperglycemia was significantly decreased compared to administration of glucose alone [39].

Human studies. Apple juice contains polyphenols such as chlorogenic acid and phloridzin, with higher levels in cloudy juice compared to clear juice. When nine healthy subjects consumed a 25 g glucose load in 400 mL of commercial apple juices, the mean plasma glucose concentrations were significantly lower at 15 and 30 min after ingestion of clear apple juice, and significantly lower at 15 min but significantly higher at 45 and 60 min after ingestion of cloudy apple juice compared to control drink [45]. The effects of apple juices on plasma glucose, insulin, GIP and GLP-1 concentrations were consistent with delayed absorption of glucose.

Berries are rich sources of polyphenols, especially anthocyanins, flavonols, proanthocyanidins and phenolic acids. In twelve healthy subjects, ingestion of sucrose (35 g) with berries (150 g of purée made of bilberries, blackcurrants, cranberries and strawberries providing nearly 800 mg polyphenols) produced a different postprandial glycemic response compared to the control without berries but with comparable profile of available carbohydrates [46]. The shape of the plasma glucose curve with reduced concentrations in the early phase and a slightly elevated concentration in the later phase indicates delayed response due to berry consumption. Berries also significantly decreased the peak glucose increment. Reduced rates of sucrose digestion and/or absorption from the gastrointestinal tract are the most probable mechanisms underlying the delayed and attenuated glycemic response. In another study, consumption of cranberry juice sweetened with high-fructose corn syrup resulted in different (but not statistically significant) pattern of postprandial glycemia compared to the similar amount of the sweetener in water [47].

In ten type 2 diabetic patients, red wine (200 mL) taken during a midday meal induced a smaller increase in blood glucose *versus* the same meal accompanied by an equivalent amount of water, with no effect of plasma insulin levels [48]. Comparable results were obtained with tannic acid, a polyphenolic component of red wine. Ethanol had no effect. In ten healthy young adults, sugar cane bioflavonoid extract reduced the postprandial glycemic response to a high-glycemic starchy meal composed of wheat biscuits and milk [49]. Ingestion of cinnamon (6 g) with rice pudding significantly lowered blood glucose response in the postprandial phase (15, 30 and 45 min) in 14 healthy subjects [50,51]. However, in another study of the same group [51], cinnamon (3 g) reduced postprandial serum insulin and increased GLP-1 concentrations without significantly affecting blood glucose response. Cinnamon has high content of proanthocyanidins.

Gastrointestinal hormone (GIP and GLP-1) profiles after consumption of 25 g glucose with coffee (400 mL containing 350 mg chlorogenic acid) indicated delayed intestinal absorption of glucose in nine healthy subjects [52]. The authors concluded that chlorogenic acid, the major polyphenol of coffee, might attenuate the intestinal glucose absorption rates and shift the site of glucose absorption to more distal parts of the intestine. In overweight men, chlorogenic acid (1 g) reduced early glucose and insulin responses during an oral glucose tolerance test [53]. Attenuated glycemic response has also been observed when sucrose (25 g) was consumed in chlorogenic acid enriched instant coffee [54].

When either 250 mL of coffee or tea was consumed with test meals, they increased the overall mean peak blood glucose concentration, but did not significantly affect the incremental area under the glucose response curve of the meals [55]. Coffee and tea contain caffeine, which increases postprandial glycemia and impairs glucose tolerance [52,56,57]. Caffeinated coffee ingested with either a high or low glycemic meal significantly impaired acute blood glucose management and insulin sensitivity compared with ingestion of decaffeinated coffee [57]. Instant black tea consumed with glucose reduced the late phase plasma glucose response with a corresponding increase in insulin [58]. The attenuation of late postprandial glycemia may be explained by an elevated insulin response following stimulation of pancreatic β-cells rather than by retarded absorption of glucose.

In conclusion, the scientific evidence on the potential of polyphenolic compounds to retard carbohydrate digestion and absorption and to suppress hyperglycemia in the postprandial state is promising. However, it is mostly based on simple *in vitro* and animal studies. Current evidence from human studies suggests that beverages such as apple juice, red wine and decaffeinated coffee as well as berries and cinnamon may improve short-term glycemic control. For substantiation of the benefits of polyphenols in the control of postprandial glucose homeostasis, more clinical studies involving subjects with normal and impaired glucose metabolism are needed. These studies should be focused on the effects of dietary polyphenols on glycemic responses induced by starch and sucrose, the main high-glycemic carbohydrates in our diet.

## 3. Influence of Polyphenols on Pancreatic β-cell Function

Insulin secretion by the pancreas involves numerous reactions which are potential targets for the action of polyphenols. Upon high blood glucose concentrations pancreatic β-cells respond to the increased demand of insulin by various mechanisms including increased insulin secretion, hypertrophy, proliferation of existing β-cells and formation of new ones from progenitor cells. The insulin release from β-cells is a cascade starting from the uptake of glucose by the GLUT2 transporters. Glucose enters a cycle of enzymatic reactions involving phosphorylation, leading to increased ATP content in the cells, and causing inactivation of ATP-sensitive potassium channels on the cell membrane. The membrane depolarizes and leads to calcium channel opening and subsequent flow of Ca^2+^ into cell. The rise in Ca^2+^ concentration promotes release of insulin by exocytosis from existing storage granules [59,60].

Prolonged hyperglycemia and hyperlipidemia, typically within development of metabolic syndrome, leads to the dysfunction of the pancreatic β-cells, reflected in autocrine insulin resistance, impaired insulin secretion, decreased expression of genes involved in insulin production and finally decrease in β-cell mass caused by apoptosis. Therefore the insulin deficiency related to metabolic syndrome in pancreas is due to both the cellular damage and the impaired efficiency in the synthesis of insulin [61].

The most extensively studied sources of dietary polyphenols in terms of pancreatic function and insulin secretion is soy, and especially its isoflavonoids, genistein and daidzein. The most commonly applied approaches in determining the effect of polyphenols on pancreatic insulin metabolism are measurement of insulin secretion or/and content in cultured pancreatic cell lines, either with or without glucose stimulation, and examination of perfused pancreas either after feeding trial/intraperitoneal injection or by directly applying the compound of interest on the isolated islets. Many of these studies, reviewed below and summarized in Tables 1 and 2, examine also the molecular mechanisms behind the observed effects of polyphenols.

### 3.1. *In Vivo* Studies with Animal Models

There are few recent studies where soy isoflavonoids at physically achievable concentrations have shown positive impact on β-cell function. Choi *et al.* [62] used genistein and daidzedin in order to study factors related to glucose and insulin metabolism using a non-obese diabetic mouse model which spontaneously develops autoimmune insulin dependent diabetes mellitus. Both isoflavonoids (0.2 g/kg genistein or daidzein for nine weeks) preserved the insulin production by the β-cells, whereas mice fed the control diet had no insulin production [62]. Another *in vivo* study performed in non-obese mice (streptozotocin (STZ) induced diabetic model) fed with fermented soybean, a Korean food ‘chungkukjang’ (5 g/100 g of diet for 6 weeks), similarly showed that the insulin concentration in pancreas was higher in the soybean- fed mice than in the non-treated control mice. In addition to enhancing the insulin production in pancreas the treatment also seemed to contribute to improved insulin sensitivity in peripheral tissues, thus necessitating smaller amounts of insulin and preventing pancreatic exhaustion [63]. The same line in results was obtained also by Lu *et al* on high-isoflavone soy protein fed STZ-diabetic rats [64].

### 3.2. Effects Observed in Cell Culture Analyses

Epigallocatechin gallate (EGCG) and rutin were examined for their ability to attenuate the glucotoxicity in rat insulinoma pancreatic β-cells (RIN m5F) [65]. The treatment increased glucose dependent insulin secretion, and was able to promote effective secretion of insulin also under chronic high glucose incubation when insulin secretion is suppressed by glucotoxicity (33 mM, 48 h), suggesting that both EGCG and rutin might preserve the glucose- sensing ability during hyperglycemia. EGCG and rutin elevated the intracellular ATP, suggesting that the increase in insulin secretion is mediated by enhancing the normal, glucose induced insulin secretion that is dependent on ATP concentrations. Interestingly, epicatechin, the precursor of EGCG, was found to inhibit insulin secretion when tested on INS-1 cells [66].

A very detailed study on the effects of dietary phenolic acids on pancreas function was carried out with cinnamic acid derivatives in INS-1 cell culture and perfused rat pancreas [67]. Among the differentially substituted cinnamic acid derivatives, the most prominent insulin releasing agents were the ones containing m-hydroxy and p-methoxy residues on the phenol ring structure, whereas cinnamic acid (no substituents in the phenol ring) was inactive. The structure promoting insulin secretion most effectively was the one of ferulic acid, containing p-hydroxy and m-methoxy structure, as it enhanced insulin secretion in a dose-dependent manner (1–100 μM), being significant already at 1 μM concentrations. Notably, the assays were performed in absence of glucose, whereas the majority of other reports have focused on glucose dependent insulin release. The results were verified also with treatment of perfused rat pancreas and intravenous administration in normal rats, where the increase in plasma insulin was detected in fasting state. Interestingly, isoferulic acid, the stereoisomer of ferulic acid did not have any effect on insulin releasing properties. This finding corroborated earlier results showing that plasma glucose lowering properties of isoferulic acid are due to increase in glucose uptake and retarding of hepatic gluconeogenesis, without any effect on pancreatic insulin output [68]

In one of the most recent studies isoflavonoids were shown to improve glucose stimulated insulin secretion in INS-1E pancreatic cell line but this effect was not due to modulation of insulin synthesis, since there was no difference on the insulin concentration in the genistein treated and non-treated cells. However, the insulin secretion upon glucose stimulation was significantly increased after 48h pre-treatment with genistein (1–5 μM). It was suggested that the effect of genistein on promoting glucose dependent insulin secretion was not mediated by the same mechanism as glucose stimulation alone, since several cellular factors related to glucose-induced insulin secretion, e.g., cellular ATP concentration, were not changed. The finding that cellular Ca^2+^ levels were elevated by the genistein treatment suggests that the improvement in insulin secretory function may be attributable to modulation of Ca^2+^ signaling and cAMP/protein kinase A (AMPK) function, but the mechanism is not yet clear [69]. The effect of genistein on insulin secretion was observable also in mouse and human pancreatic islets showing non-species-specific and biologically relevant effect.

Also numerous other publications report on the insulin secretagogic activities of dietary phenolics e.g., anthocyanidin and anthocyanin compounds in INS-1 cell line [70], aspalathin, component from rooibos tea *Aspalathus linearis*, in RIN-5F cells [71], and compounds isolated from *Eriobotrya japonica* in INS-1 Cells [66].

There are also indications for the function of polyphenols on β-cells by other mechanisms besides affecting insulin secretion. Ethanol extracts from the root, stem, leaf and fruit of the Canadian lowbush blueberry *Vaccinium angustifolium*, a very rich source of flavonoids, were analyzed for insulin secretagogue and proliferative effects [72]. The insulin secretion was measured from growth arrested (tetracycline-treated) β-cells in order to distinguish the insulinotropic effect from the cell proliferative effect. Only slight enhancement was observed in the glucose stimulated insulin secretion with the treatment by leaf and stem extracts, but the effect on the cell proliferation rate was found to be significantly increased by the treatment with the fruit extract when compared to vehicle-only control, suggesting a potential capability to restrain β-cell damage in metabolic syndrome.

Another study showing β-cell protective effect of flavonoids was performed by mixtures of flavonoids quercetin, luteolin and apigenin in RINmF5 cells [73]. Flavonoids showed anti-inflammatory action in a treatment with interleukin 1β (IL-1β) and interferon γ (IFN- γ), and the effect was verified at transcriptional analysis of inflammation-related genes, suggesting a role for flavonoids in the restoration of insulin secretion capacity by preventing the cytokine-induced β-cell damage.

### 3.3. Effects Observed in Isolated/Perfused Pancreas

Oral administration of rutin (100 mg/kg, 45 days) was shown to promote β-cell viability in STZ induced diabetic rats [74]. It was suggested that the β-cell restoring effect of rutin was due to enhanced ability to scavenge free radicals and mediate antioxidant enzyme activity in the pancreas. Similarily, quercetin, the aglycon molecule of rutin, showed β-cell restoration when used as dietary supplement (0.5% of diet for 14 days) in STZ induced diabetic mice [75]. Gene expression analysis showed that quercetin restored the cell proliferation capacity inhibited by STZ treatment, and resulted in higher plasma insulin levels. In addition oxidative stress markers were reduced in pancreas, further ameliorating the oxidative damage associated with diabetes. Quercetin has been studied also in STZ-diabetic rats by intraperitoneal injection, and the preservation of islet cells and restoration of insulin production has been observed in two studies [76,77].

Intraperitoneal injection of (−)epicatechin in alloxan treated mice demonstrated β-cell- regenerative capacity [78]. Similarily, (−)epicatechin or quercetin promoted increased release of insulin when isolated rat islets were exposed to them, whereas naringenin and chrysin inhibited it [79]. Additional observations with dietary sources of polyphenols include the protection of non-obese diabetic mice pancreatic islets from infiltration of immune cells and induction of insulitis by feeding grape powder and high vitamin A supplement [80]. An interesting approach was taken to study olive mill waste which is a rich source of phenolic compounds, especially phenylethanol compound hydroxytyrosol. Fractions of olive mill waste were studied for a range of hypoglycemic and antioxidative effects, including the effect in insulin secretion in alloxan- induced diabetic rats administered by intraperitonial injection. Mainly the purified hydroxytyrosol fraction showed protective action on alloxan-damaged β-cells [81].

Phytochemical- rich extracts from other than dietary plants have also been studied for their impact on pancreatic insulin production and release. Studies have focused especially on medicinal plants known for their anti-diabetic effects. Seed extracts of *Eugenia jambolana* enhanced insulin secretion from isolated islets of STZ-induced diabetic rats in the presence of 10 mM glucose [82]; eupatilin, a flavone from *Artemisia princes*, elevated pancreatic insulin concentration in type 2 diabetic mouse model (db/db) [83]; and aqueous extract from *Abutilon indicum*, a plant used as traditional medicine in Thailand, stimulated insulin secretion from isolated rat islets and INS-1E cells [84]. A fraction containing apigenin and rutin from *Teucrium polium*, a medicinal plant from Iran, mediated insulin secretion increase in the presence of STZ on isolated rat pancreatic islets [85].

In conclusion, it is obvious that the pancreas is one of the targets of dietary polyphenol bioactivity, as several of the studied plant extracts and purified compounds exhibit beneficial effects on β-cell function and insulin release in different diabetic models. However, no single mechanism has been identified to be responsible for the response. For instance, in INS-1E cells genistein did not increase the level of intracellular ATP upon the glucose stimulation, whereas treatment of the RIN-m5F cells with EGCG and rutin elevated the ATP level [65]. This suggests that the latter treatment enhanced the signaling route mediated normally by glucose, whereas the genistein treatment had effect on alternative mechanism of insulin secretion. A range of different compounds and plant food extracts studied show various activities relevant for insulin secretion, and the activities are different on normoglycaemic controls and the subjects with symptoms of metabolic syndrome. The different effects of various molecules were highlighted in a study showing that even small changes (e.g., hydroxylation) on the molecular backbone result in different insulin- releasing capacity [67]. The studies have been made mainly using cell cultures and animal models, and motivate to proceed to human controlled trials.

## 4. Influence of Polyphenols on Tissue Uptake of Glucose

Dietary polyphenols may also influence glucose metabolism by stimulating peripheral glucose uptake in insulin-sensitive and non-insulin sensitive tissues. Glucose transport pathways can be classified either as insulin or non-insulin mediated pathways. Non-insulin mediated glucose uptake takes place in all tissues and is responsible for the basic glucose transport into the cells in post-absorptive state. In contrast the insulin mediated glucose uptake takes place only in insulin sensitive tissues. Insulin stimulates the glucose uptake in skeletal muscle, which is the largest site for disposal of dietary glucose, and in adipose tissue, whereas in the liver it decreases the hepatic glucose output rate by increasing the storage of glucose as glycogen.

Glucose uptake is mediated by the action of glucose transporters (GLUTs) on the cell surface [86]. It is important to point out that among the 13 GLUTs identified so far [87], only GLUT4 is an insulin sensitive glucose transporter. Based on sequence comparison, the GLUT isoforms can be grouped into three classes. Class I comprises GLUT1–4; class II, GLUT6, 8, 10, and 12 and class III, GLUT5, 7, 9, 11 and H^+^-myo-inositol cotransporter (HMIT) [88]. Tissue- and cell-specific expression of the well-characterized GLUT isoforms underlies their specific role in the control of whole-body glucose homeostasis. Numerous studies with transgenic or knockout mice support an important role for these transporters in the control of glucose utilization, glucose storage and glucose sensing, but more studies are needed to elucidate the mechanisms behind.

Glucose transporters from class I are actively involved in glucose mobilization and uptake. GLUT1 and GLUT3 are responsible for maintaining the basal glucose uptake, and contrary to GLUT4 are abundant in several tissues [89]. GLUT1 is widely distributed in fetal tissues and it is expressed at high levels in erythrocytes and endothelial cells of barrier tissues in adults, while GLUT 3 is mostly expressed in neurons and placenta. Glucose is transported into and out of liver cells by the concentration-driven GLUT2 [90], which is also expressed by renal tubular cells, small intestinal epithelial cells that transport glucose and pancreatic beta cells. GLUT4 is expressed by muscle, adipose and kidney cells and remains stored in insulin-responsive compartments within the cells until insulin mediates its localization on the cell surface.

The most studied insulin signalling pathway leading to increased muscle glucose uptake involves binding of insulin to GLUT4, phosphorylation of downstream insulin receptor substrates (IRS) and activation of several signalling enzymes such as phosphatidylinositol-3 kinase (PI3K) and Akt-serine/threonine kinase. The cascade promotes GLUT4 glucose transporter translocation from an intracellular pool to the plasma membrane [91,92]. In addition to PI3K activity, there are also other signalling routes involved in the cellular response to insulin stimulation and a detailed overview of the basic insulin signalling and regulation of glucose metabolism was reviewed some years ago by Saltiel and Kahn [93]. In this sense, a molecular mathematical model of glucose mobilization and glucose uptake has been recently developed considering the kinetics of GLUT2, GLUT3 and GLUT4, the process of glucose mobilization by glycogen phosphorylase and glycogen synthase in liver, as well as the dynamics of the insulin signalling pathway [90].

Among the potential compounds stimulating glucose uptake, several foods and plant extracts rich in polyphenols have been the object of extensive research during the last years (Tables 1 and 2). The methods most commonly used to study the effects of phenolic compounds on peripheral glucose uptake are cell culture assays in rat skeletal muscle (rat L6 myotubes) and adipose (3T3-L1) cell lines. Most studies reported in the literature so far base their glucose uptake mechanisms in insulin mediated pathways, mainly cAMP/protein kinase A (AMPK) and PI3K activation. The insulin-stimulated glucose uptake shows to be dose-dependent in most cases.

### 4.1. Effects of Pure Compounds on Glucose Uptake

Chlorogenic acid and ferulic acid caused a modest, but significant increase in 2-deoxy-d-glucose transport into L6 myotubes, showing comparable performance to metformin and 2,4-thiazolodinedione, two common commercial oral hypoglycemic drugs [94]. Purified aspalathin from green roiboos extract increased dose-dependently and significantly glucose uptake by L6 myotubes at concentrations 1–100 μM, irrespective of insulin absence [71]. As aspalathin is capable of scavenging intracellular reactive oxygen species (ROS), its antioxidative function may be involved in the stimulation of glucose uptake and insulin secretion, and hence glucose homeostasis. An inhibitory effect of EGCG was observed in L6 skeletal muscle cells on insulin resistance induced by dexamethasone, a glucocorticoid [95]. A 24 h- treatment with EGCG attenuated the effect of dexamethasone on glucose uptake and improved insulin-stimulated glucose uptake in a dose-dependent manner by increasing GLUT4 translocation to plasma membrane [95]. EGCG was able to increase the phosphorylation of AMPK, suggesting that the AMPK signalling pathway is likely responsible for the EGCG-stimulated GLUT4 translocation.

Resveratrol increased glucose uptake in C2C12 skeletal muscle cells by activating AMPK [96]. In the absence of insulin, the effect of resveratrol on glucose uptake was primarily dependent on AMPK activation, without involving PI3K. In the presence of insulin, resveratrol also potentiated the effect of insulin on glucose uptake *via* AMPK activation, but leading to activation of the PI3K-Akt signal pathway [96]. Resveratrol treatment during 15 weeks increased both insulin-stimulated whole-body and steady-state glucose uptake of both soleus muscle and liver in high cholesterol-fructose-fed rats [97]. It enhanced membrane trafficking activity of GLUT4 and increased phosphorylation of IR in insulin-resistant soleus muscles. Interestingly the activation of estrogen receptor seems to be crucial for resveratrol-stimulating muscular glucose uptake *via* both insulin-dependent and –independent pathways [97]. Additional putative function for resveratrol was found in a study reporting that Akt/protein kinases B (PKB) and GLUT4 or GLUT1 translocation is not involved in resveratrol activation. The mechanism seems to involve sirtuin-dependent AMPK activation that may lead to stimulation of the intrinsic activity of GLUT4 [98]. Sirtuins are a family of histone/protein deacetylases, among which, SIRT1 has been suggested to play a role in regulating glucose homeostasis and may be involved in the insulin signalling cascade [99,100].

Kaempferol and quercetin isolated from the traditional Chinese medicine *Euonymus alatus* improved glucose uptake of insulin stimulated 3T3-L1 mature adipocytes and had no effects on GU without insulin [101]. The results indicated that both flavonoids could ameliorate insulin resistance peripherally, similar to a PPARγ agonist such as rosiglitazone. Kaempferol 3-neohesperidoside, a flavonoid glycoside isolated from Cyathea phalerata, stimulated glucose uptake in rat soleus muscle mainly *via* the PI3K pathway [102]. Another kaempferol derivative, kaempferitrin (3,7-dirhamnoside), has recently been shown to inhibit GLUT4 mediated glucose uptake in differentiated 3T3-L1 cells by interfering with insulin signaling pathway and also by directly interacting with membrane GLUT4 [103]. Contradictory, at the same time other authors have found opposite results for kaempferitrin treatment of the same cell line, demonstrating increase in the glucose uptake [104]. The latter results agreed with the glucose uptake stimulation by kaempferitrin found in rat soleus muscle [105]. This suggests that the effect of kaempferitrin on insulin mediated glucose uptake might be a cell type specific function. Inhibitory effect on glucose uptake has been observed in adypocyte cells also by the isoflavone genistein with concentrations 20–50 μM [106].

### 4.2. Effects of Polyphenol Containing Foods and Plant Extracts on Glucose Uptake

Several plant based foods and extracts have been reported to enhance glucose uptake *in vitro.* A green tea polyphenolic extract was reported to regulate the expression of genes involved in glucose uptake and insulin signalling pathways in the muscle tissue from rats with metabolic syndrome induced by a high fructose diet [107]. The tea extract significantly increased the mRNA levels of GLUT4 in the muscle. A procyanidin extract from grape seed has been reported as an insulinomimetic agent since it stimulates glucose uptake in 3T3-L1 adipocytes and L6E9 muscle cells *via* PI3K – pathway [108]. A more detailed study with same approach showed recently that the grape seed extract interacts with the insulin receptor inducing its phosphorylation and consequently leading to increased glucose uptake *via* pathway requiring Akt. However, the treatment leads to differential phosphorylation of the insulin signalling pathway proteins than insulin does [109].

Fruit juice extract of *Momordica charantia* (bitter melon) was shown to stimulate glucose and amino acid uptakes into L6 muscle cells in a similar manner to insulin [110]. Pharmacological concentrations had inhibitory effects, while physiological concentrations had insulin-like stimulating effects, a finding that points out the importance of the concentration of the bioactive compounds in stimulating glucose uptake into muscle cells. Water-soluble components in bitter melon also enhanced the glucose uptake at sub-optimal concentrations of insulin in 3T3-L1 adipocytes, which was accompanied by an increase in adiponectin secretion [111]. Charantin, steroid, glycosides, flavonoids and their derivatives may in part be responsible for the observed up-regulatory activities of glucose uptake and mRNA expression of GLUT4, PI3K and PPARγ in bitter melon extracts but more research is needed to confirm this statement [112]. Another study on the effect of fruit juices on the glucose uptake was performed with blueberry juice. The biotransformation of the juice with a novel strain of bacteria isolated from the blueberry flora (*Serratia vaccinii)* increased its phenolic content and antioxidant activity [113] and modified its biological activity [72]. The juice extract increased AMPK phosphorylation and glucose uptake in both muscle cells and adipocytes, but it also inhibited adipogenesis [114].

Common spices, such as cinnamon, cloves, turmeric and bay leaves also show insulin-like activity *in vitro* [115]. For instance, cinnamon polyphenols with doubly linked procyanidin type-A polymers appear to be unique for their insulin- like activity [115]. A water-soluble cinnamon extract showed to increase the activity of autophosphorylation of the IR and decrease the activity of tyrosine phosphatase *in vitro* [116]. The mechanism of cinnamon’s insulin-like activity may be in part due to increases in the amounts of IRβ and GLUT4 [117]. *In vivo* insulin-regulated glucose utilization was also enhanced by cinnamon extracts by increasing glucose uptake in rats with insulin resistance induced by a high-fructose diet [118,119].

Several plant extracts from plants used in traditional medicine have been as well reported to promote insulinotropic / insulinomimetic activities. Four isoflavonoids (genistein-derivatives), recently identified from a branch extract fraction of the Vietnamese traditional herb *Tetracera scandens*, exhibited significant glucose uptake activity both in basal and insulin-stimulated skeletal muscle cells in a dose-dependent manner. AMPK activation and GLUT4 and GLUT1 expressions appear to be involved in the glucose uptake stimulation mechanism [120]. A recent review has also reported that penta-galloyl-glucose (PGG), a polyphenolic compound highly enriched in a number of medicinal herbals, exhibits multiple biological activities relevant in diabetes prevention [95]. Both β-PGG and its anomer α-PGG have showed insulin-mimicking activity in the absence of insulin, and α-PGG was more potent than β-PGG [121]. α-PGG itself stimulated glucose uptake in 3T3-L1 adipocytes. However, α-PGG weakened the activity of insulin if treated together. α-PGG induced phosphorylation of the IR, PI3K and Akt, and stimulated membrane translocation of GLUT4. Plant root extracts can also exert glucose uptake enhancement properties. For example, the aqueous extract of *Canna indica* root (Cannaceae), rich in flavonoid compounds, caused a dose- and time- dependent induction of glucose uptake activity in L8 muscle cells [122]. The authors suggested that GLUT1 protein synthesis and the activation of PI3K are critical for the increase in glucose transporter activity at the plasma membrane.

In conclusion, insulin stimulates glucose uptake in skeletal muscle and adipose tissue primarily by eliciting the translocation of GLUT4 from an intracellular pool to the plasma membrane [123]. Current data suggest that polyphenols mainly affect glucose transport and insulin-receptor function, both of which play essential roles in diseases related to carbohydrate metabolism [124]. To date glucose uptake data from polyphenols mainly derives from animal cell culture studies. The most likely mechanism implies the PI3K activity signaling route. Recent studies use amounts of phenolic compounds closer to physiological range. However, doses of relevance to human health are still unknown, and deserve further research.

## 5. Influence of Polyphenols on Liver Function to Maintain Glucose Homeostasis

Liver plays a major role in the regulation of blood glucose levels in tight cooperation with peripheral tissues. As estimated, liver is responsible of taking up one third of the postprandial glucose [125], and stores effectively glucose as glycogen *via* glycogenesis. In fasted state, liver is the main regulator of maintaining stable blood glucose levels and produces glucose by two different routes either by breaking down glycogen (glycogenolysis) or by synthesising glucose from other metabolites such as pyruvate, lactate, glycerol and amino acids (gluconeogenesis). The key enzymes responsible for the regulation of glycogenesis are glucokinase (GK) and glycogen synthase (GS). Pyruvate carboxylase, phosphoenolpyruvate carboxykinase (PEPCK), fructose-1,6-bisphosphatase, and glucose-6-phosphatase are the major enzymes responsible of the regulation of gluconeogenesis [126].

Several factors influence hepatic glucose homeostatic control. At hormonal level insulin and glucagon directly regulate hepatic glucose metabolism. For instance, in fed state insulin suppresses liver glucose production and output *via* insulin receptor pathway [127]. Furthermore, the central nervous system mediates part of the effects of insulin and of other signals such as long chain fatty acids (LCFAs) to exert higher control on hepatic glucose metabolism [128,129]. In type 2 diabetes and insulin resistant state the control of hepatic glucose metabolism and hepatic glucose output are disturbed, and the inability of the liver to respond to insulin results in severe defects in the regulation of glucose homeostasis such as increased hepatic glucose output and hyperglycemia. Non-alcoholic hepatic steatosis, the accumulation of triglycerides in the liver that might lead to fibrosis, is clearly associated with hepatic insulin resistance. However, it is not clear whether insulin resistance causes the excessive accumulation of triglycerides (TG) in liver, or whether the increase in TG itself plays a causal role in the development of hepatic or systemic insulin resistance [130]. In mice, a high-fat diet has been shown to first deteriorate hepatic insulin sensitivity in association with hepatic accumulation of short to medium chain fatty acylcarnitines, prior to affecting peripheral insulin sensitivity [131]. Several studies indicate improved liver glucose and lipid metabolism in normal, obese and diabetic mouse or rat models after treatment with different polyphenol-rich diets. The following section discusses the potential mechanisms of effects of polyphenols on glucose metabolism in liver.

### 5.1. Effects of Green Tea and Epigallocatechin Gallate (EGCG)

Tea catechins and their effects on liver glucose metabolism have been effectively studied in animal and cell culture models. Green tea extracts and green tea catechins such as epigallocatechin alone have been shown to decrease blood glucose levels and concomitantly also liver triglyceride contents. In streptozotocin-induced diabetic rats oral administration of EGCG (25 mg/kg b.w./day) for eight weeks significantly alleviated the increase in serum glucose levels and serum TG levels [132]. However, the study did not include any tissue specific analyses. Supplementation of the diet with 0.5% and 1.0% green tea for six weeks reduced liver TG concentrations 27–30% in fructose-fed ovariextomized rats as compared to fructose and starch fed control diets [133]. Several other studies have also shown reduced blood glucose levels and liver TG contents after feeding with green tea or EGCG. For instance, supplementation of high-fat diet (60% energy as fat) fed mice with dietary EGCG (3.2 g/kg diet) for 16 weeks resulted in decreased blood glucose levels and decreased liver TG contents [134].

The potential mechanisms explaining how liver could contribute to the reduced blood glucose levels in green tea and EGCG treated animal models have been studied as well. Wolfram *et al*. [135] assessed glucose and insulin tolerance in db/db mice and investigated the effect of 5–7 weeks EGCG supplementation on gene expression in liver tissue using real-time quantitative PCR (RT-PCR). EGCG supplementation (2.5–10.0 g/kg) resulted in decreased blood glucose levels in a dose dependent manner as tested by OGTT. In the fed state plasma glucose, free fatty acid and TG levels were lower and insulin levels higher in EGCG-treated db/db mice than in control mice. EGCG treatment increased the expression of liver glucokinase (glycogenic enzyme), carnitine palmitoyl transferase-1β and decreased the expression of gluconeogenetic enzymes phosphoenolpyruvate carboxykinase (PEPCK). The authors suggested that the potential mechanisms to explain the T2DM amelioration by the dietary supplementation of EGCG could be the reduced endogenous liver glucose production and increase in glucose-induced insulin secretion [135]. Furthermore, DNA microarray analysis of H4IIE rat hepatoma cells exposed to EGCG (50μM), showed that genes involved in the synthesis of fatty acids, triacylglycerol, and cholesterol were strongly downregulated, also genes involved in gluconeogenesis were downregulated whereas genes involved in glycogenesis were upregulated.

These findings are in line with cell culture studies that have shown reduced hepatic gluconeogenesis and glucose output after exposure to EGCG or green tea extract [136,137]. For instance, Collins *et al*. [136,137] studied the role of EGCG in hepatic gluconeogenesis using isolated hepatocytes exposed to physiologically relevant concentrations of EGCG (≤1 μM). EGCG decreased glucose production by inhibiting expression of the gluconeogenetic enzymes (PEPCK and glucose-6-phosphatase) in a similar manner to insulin. However, EGCG was not found to activate the insulin signalling pathway. Further tests showed that EGCG activated AMPK, which was shown to be necessary for the observed inhibition of gluconeogenetic enzyme expression. AMPK activation was mediated by the calmodulin-dependent protein kinase kinase CaMKK [136]. Furthermore, ROS production induced by EGCG was shown to be required for the activation of AMPK and inhibition of gluconeogenesis. The study by Collins *et al*. showed that EGCG exerts toxic effects on primary hepatocytes already at concentration of 10 μM. Other studies have found EGCG to have similar effects on hepatic glucose metabolism, though with concentrations exceeding 10 μM [137,138].

### 5.2. Effect of Soy Isoflavones, Genistein and Daidzein

Similarly to green tea also soy and soy isoflavones genistein and daidzein supplementation (0.2 g/kg) have been found to decrease blood glucose levels and to reduce liver TG concentrations in db/db mice model [139] and in non-obese diabetic mice [62]. Both studies found reduced glucose-6-phosphatase and PEPCK liver activities and increased glucokinase activities suggesting that genistein and daidzein suppresses liver glucose output. Cederroth *et al*. [140] studied the mechanisms behind the effects of soy supplementation rich in equol, daidzein and genistein, in normal CD-1 mice. Phytoestrogen- rich supplementation (198 ppm daidzein and 286 ppm genistein equivalents in the high phytoestrogen diet) from conception to adulthood was found to activate AMPK in liver but also in white adipose tissue and muscle. The authors hypothesised that high-phytoestrogen-fed mice would have altered mitochondrial metabolism and found that the expression of peroxisome proliferator-activated receptor α (PPARα) and its coactivator peroxisome proliferator-activated receptor γ coactivator (PGC-1α) were upregulated in liver, white adipose tissue and muscle suggesting improved fatty acid β-oxidation [140]. Potentially, in normal (non-obese) mice activation of PPARα could lead to change from glucose utilization to fatty acid oxidation to produce fuels, instead of creating new TGs [141]. Increased fatty acid β-oxidation might protect against non-alcoholic hepatic steatosis and therefore could also improve insulin sensitivity and glucose metabolism in liver. Furthermore, decrease in hepatic TG pools has been shown to correlate with improved insulin sensitivity [130]. However, the role of TGs in the development of insulin resistance is not yet clear. Chungkukjang (a fermented soybean food) supplementation also resulted in significantly higher hepatic GK activity and decreased activity of gluconeogenic enzymes G6Pase and PEPCK in db/db mice when compared to control group [142]. However, also insulin secretion was improved after Chungkukjang supplementation.

### 5.3. Effect of Citrus Flavonoids, Grape Polyphenols and Phenolic Acids

The citrus flavonoids, hesperidin and naringin (0.2 g/kg) were shown to lower blood glucose levels as compared to the control diet fed to db/db mice [143]. Similarly to green tea and soy, hesperidin and naringin also significantly reduced plasma free fatty acid, TG and total cholesterol levels in plasma as well as hepatic TG content. These physiological changes were postulated to be due to increase in hepatic glucokinase mRNA, decrease in expression of the gluconeogenetic enzymes PEPCK and G6Pase, and improvement in lipid metabolism caused by altered activities of hepatic lipid metabolizing enzymes [143,144]. Furthermore, naringenin (25–100 μM), the aglycone form of naringin, was shown to suppress hepatic glucose production from hepatoma cells in a dose dependent manner even though naringenin did not have any impact on gluconeogenetic gene expression [145]. However, naringenin exposure led to decrease in cellular ATP levels.

Grape seed-derived polyphenols such as procyanidins have been also shown to alleviate insulin resistance in mice fed with high-fat diet. Simultaneous supplementation of grape-seed derived procyanidin-rich extract and *G. pentaphyllum* extract (altogether 80 mg/kg) improved glucose tolerance and HOMA-IR index, as well as lowered the high-fat-diet induced serum glucose levels and also increased the activity of hepatic glucokinase [95].

Unlike polyphenols discussed above, resveratrol was shown to have opposite effects and increase the expression and activity of gluconeogenetic enzymes. As Ganjam *et al*. [146] showed, rats treated with resveratrol (5–10 mg/kg/day) by intraperitoneal injections for 2 days lead to decreased GK mRNA levels in liver in a dose-dependent manner. The decreased GK mRNA expression was accompanied by a reduction in GK protein levels. In primary rat hepatocyte cultures resveratrol (10–50 μM) also suppressed GK expression and conversely enhanced PEPCK expression. The suppression of GK by resveratrol was found to be mediated, at least partly, by the deacetylation of FoxO1-transcription factor and further binding to HNF-4 (hepatocyte nuclear factor) that can restrain it from its binding site in the proximal GK promoter [146]. Similar effects of resveratrol on hepatic glucose metabolism have been shown with H4IIE rat hepatoma cells [147]. However, there are controversial findings showing that activation of SIRT1 was repressing forkhead transcription factors including Foxo1 in different cell models [148]. On the other hand, the results are supported by the fact that knockdown of SIRT1 in liver leads to decrease in gluconeogenesis [149]. The roles of Foxos and sirtuins in the regulation of hepatic glucose metabolism clearly need further clarification.

Administration of phenolic acid fraction of rice bran containing considerable amounts of *trans*-cinnamic acid derivatives (ferulic acid, and *p*-coumaric acid) and ferulic acid alone for 17 days was shown to exert hypoglycemic effects and to elevate liver glycogen synthesis and glucokinase activity in db/db mice compared with the control group [150]. Insulin secretion was also improved, and it was postulated that the rice bran fraction and ferulic acid could have increased insulin action and the utilization of dietary glucose in the liver.

In conclusion, several different polyphenol classes have been shown to reduce hepatic glucose output by suppressing gluconeogenetic enzyme expression and increasing the activity of glucokinase to improve glycogenesis and glucose utilization. EGCG has been shown to exhibit these effects by activating AMPK. Furthermore, theaflavins have been shown to activate AMPK in HepG2 cells and to attenuate hepatic lipid accumulation [151]. Activation of AMPK by dietary polyphenols leads to suppression of hepatic gluconeogenesis and induction of fatty acid β-oxidation that both improve hepatic glucose utilization and insulin sensitivity [152]. Resveratrol seems to function in opposite way by activating FoxO1 and inducing hepatic gluconeogenesis. In contrast, resveratrol has been also shown to activate hepatic AMPK [153,154]. Therefore the role of resveratrol in hepatic glucose metabolism needs further clarification. Recent findings suggest that FoxO1 integrates insulin signalling with hepatic mitochondrial function and inhibition of Foxo1 can improve hepatic metabolism during insulin resistance and the metabolic syndrome [155]. In addition to the changes in hepatic glucose utilization and output, most of the *in vivo* studies report changes in hepatic TG contents as well as in blood TG contents. As the hepatic lipid accumulation is connected to insulin resistance it is therefore possible that phytochemicals could exert their effects indirectly on hepatic glucose output by influencing lipid metabolism. *In vivo* studies have also reported increased insulin secretion or changes in blood insulin levels after polyphenol rich diet and therefore the hepatic effects could be also due to insulin signalling.

## 6. Impact of Polyphenols on Maintenance of Glucose Homeostasis

The majority of the studies on the effects of dietary polyphenols on carbohydrate homeostasis are performed by specific assays focusing on certain parts of the regulatory system. There is, however, increasing data from long-term dietary studies on polyphenol supplementation in animal models and in humans. In such trials, the most common outcome parameters are the blood glucose and insulin levels, the measurements of body fat composition and circulating levels of triglycerides, free fatty acids or other lipid metabolism related biomarkers such as cholesterol, measurement of inflammatory markers, and factors related to the redox status of the organs. The most relevant mechanisms underlying the beneficial health effects are, however, difficult to postulate as the molecular mechanisms have not been comprehensively studied.

### 6.1. Evidence from Epidemiological Studies

In epidemiological studies, very few of the individual polyphenolic compounds alone have been so far demonstrated to have a beneficial effect on prevention of type 2 diabetes. A prospective study of flavonoid intake from the Finnish diet concluded that quercetin and myricetin are associated with reduced risk of type 2 diabetes [5]. On contrary, intakes of quercetin, kaempferol, myricetin, apigenin, and luteolin were not associated with reduced risk of type 2 diabetes in The Women’s Health Study [156]. However, the inverse association with diabetes risk in epidemiological studies has been shown with whole polyphenol-rich diets/food items, which suggests that the effects of polyphenols on disease risk cannot be attributed to single compounds. This is an important issue for consideration for the mechanistic studies using *in vitro* models. Whole grain rich diets have been linked with decreased risk of obesity and type 2 diabetes in epidemiological studies [157,158], and high coffee consumption has been associated with lower prevalence of metabolic syndrome [19,159]. Apples and tea consumption have also been linked to lowered incidence of type 2 diabetes in middle-aged women [156], and apples and berries were the most important contributors lowering the risk in Finnish men and women [5]. In a meta-analysis including nine cohort studies with follow-up ranging from 5 to 18 years, tea consumption was associated with prevention of development of type 2 diabetes [160]. Over four cups of tea per day was required to produce the beneficial effect, although also smaller intake has been shown to be effective in lowering the risk of obesity and blood glucose levels [161]. These beneficial effects by both coffee and tea intakes have been demonstrated also in a recent cohort study where the effect of single compounds magnesium, potassium, and caffeine alone was excluded, and it also was concluded that the effect was not mediated by blood pressure lowering effect [162].

### 6.2. Evidence from Clinical Trials

There are only a few controlled interventions studying the effects of specific polyphenols or food products in amelioration of the symptoms of the metabolic syndrome. One of the polyphenols studied most frequently *in vitro* is EGCG and/or its source green tea extract. In spite of promising results from animal and *in vitro* testing, EGCG treatment has not been shown to improve insulin resistance in humans, although some beneficial health impacts have been observed [163,164]. One study on type 2 diabetes patients showed increased levels of insulin after 12 weeks of diet supplemented with catechin-rich (582.8 mg) green tea [165], and another study revealed correlation between high intake of tea polyphenols and improved insulin levels in type 2 diabetes patients [164]. In contrast to tea, interesting results have been produced in human trials by dark chocolate consumption. Dark chocolate (100 g dark chocolate bar containing approximately 500 mg of polyphenols for 15 days) improved insulin sensitivity along with reducing blood pressure in healthy subjects [166] and similar results were reported with the same treatment on hypertensive subjects [167]. However, consumption of a flavanol-rich cocoa drink (150 mL twice a day, approximately 900 mg flavanols) for 15 days did not improve insulin resistance or blood pressure in individuals with essential hypertension [168]. Grape seed extract given to type 2 diabetic patients for 4 weeks, had positive effect on several inflammatory markers and glycaemia, but did not result in statistically significant changes in HOMA-IR [169]. In regard of whole grain consumption the beneficial health effects may also be, at least partly, due to the polyphenol content of whole grain products, as a polyphenol-rich wheat bread had higher glucose lowering and antioxidative effect than a control wheat bread during a 9-day study period [170]. Other promising plant food candidates with diabetes preventive potential include cinnamon, bitter melon and fenugreek [171].

### 6.3. Evidence from Animal Experiments

On the other hand, considerable evidence is available on the effects of several polyphenols and polyphenol-rich food items in ameliorating insulin resistance and improving insulin sensitivity in experimental animals. In mice fed high-fat diet indications towards beneficial effect in glucose/insulin signaling have been obtained by catechin [172], EGCG [134], grape seed procyanidins [173], and blueberry [174]. Similarly in rats fed high-fat diet isoflavones [175], quercetin [176], and blueberry [177] have alleviated the markers of metabolic syndrome. Another type of high-calorie diet, fructose-rich diet, has been applied in rat experiments, producing promising results in balancing the glucose/insulin metabolism with myricetin [178], fenugreek seed extract or quercetin [179], longan flower extract [180], green tea [181] and cinnamon [119,179]. Moreover, insulin sensitivity was improved in the CD-1 mice that have genetic susceptibility to obesity and type 2 diabetes by feeding a diet containing soy [140].

In conclusion, the evidence from epidemiological studies on the protective role of polyphenol-rich foods against development of type 2 diabetes is suggestive, but in spite of the large array of studies *in vitro* and the positive results in animal models, only a handful of controlled human interventions confirm these results. The discrepancy between the results from animal and human studies may be due to species specific differences, but also other factors such as genetic variability and general study set-up (dosage of supplementation, number of study objects, length of intervention) most likely have an impact on the outcome. It is clear that more tightly controlled human studies should be conducted in order to draw conclusions about the role of polyphenols in insulin resistance.

## 7. Conclusions and Future Prospects

Foods or meals high in available carbohydrate such as starch or sucrose induce hyperglycemia and hyperinsulinemia. Regular consumption of diets with high glycemic impact may increase the risk of obesity, type 2 diabetes and cardiovascular disease by promoting excessive food intake, pancreatic β-cell dysfunction, dyslipidemia, and endothelial dysfunction [27]. The potential of polyphenols in controlling glycemia is a very intensively studied area, encompassing a large piece of scientific literature; studies listed in PubMed in this field in 2009 alone gave over 70 hits. Indications for positive effects of a large number of polyphenols on glucose homeostasis have been obtained *in vitro* and in animal studies, but definitive conclusions, especially from controlled human studies and at the molecular mechanistic level have not been obtained. There is a shortage of human studies with clinically relevant end-points indicating effects during postprandial handling of dietary carbohydrates, pancreatic insulin secretion and its functions on glucose homeostasis in peripheral tissues.

The field is broad because carbohydrate metabolism constitutes one of the most important physiological functions in the human body involving numerous different organs, tissues and cell types. On the other hand, the amount of dietary constituents potentially contributing to glucose homeostasis is vast, and especially for bioactive non-nutrients, such as polyphenols, mostly unidentified. One important issue in research on dietary phytochemicals is the lack of knowledge on their absorption, metabolite composition and tissue distribution. Plants contain thousands of metabolites in different quantitative and qualitative combinations, and the identification of combinations of active molecules in a given metabolic pathway is an extremely challenging task.

The studies performed in cell cultures with single plant phenolic compounds at concentrations exceeding pharmacological doses do not have much predictive value of the effects these compounds would produce when fed in diet and harnessing their target tissues after the metabolism of gut microbiota and the human organs. It is therefore understandable that data from controlled human interventions is missing or contradictory in spite of the positive epidemiological evidence with e.g., whole grains, apples, tea and coffee, and studies with pure compounds and extracts showing effects in various steps of glucose metabolism in cell and animal models. However, in comparison to the studies reported a decade ago, the current *in vitro* studies tend to use amounts of phenolic compounds closer to the range of physiological levels than pharmacological doses.

It is obvious that more human trials with well defined diets and controlled study set-ups should be made to test the hypotheses created by the mechanistic studies, and early biomarkers are needed to reveal the effects of subtle dietary changes in intervention studies. Dose response studies and pharmacokinetic profiling of the hypothetic active metabolites should also be made. More focus should be laid on the studies analysing the effect of whole plant/food extracts in order to follow the synergistic bioactivity of the different phytochemical compounds present in the food concomitantly. Also the interplay between the phenolic compounds and other food constituents such as fibre, is an interesting topic that undoubtedly deserves attention in the case of food products that are rich in both polyphenols and fibre, including whole grain products and fruits like apple.

The research on health effects of plant-based foods will benefit from taking holistic approaches with the aim to resolve an array of effects mediated by an array of bioactive metabolites on the whole body level. One of the key factors will be the combination of the different omics-profiling techniques in the concept of systems biology, or nutrigenomics as termed in the context of nutrition related sciences. Whilst transcriptomics and proteomics characterization are already available on relatively routine laboratory analyses, metabolomics analyses are also rapidly developing, and are expected to be an even more useful tool in making the link between food constituents and subsequent clinical outcome, also in diabetes related research [182]. Especially the non-targeted profiling assays where the metabolite pools of control group and test group (e.g., after dietary challenge) are compared and the metabolite signals significantly differing are resolved with statistical analysis methods. In the elucidation of the effects of dietary phytochemicals on human health, such analyses will likely play a key role in pointing out the factors from bioavailability, absorption, microbial metabolism, whole body distribution, tissue localization and mechanisms of action that would not be achievable by targeted single compound assays.

**Main conclusions**

- There are indications for positive effects on glucose homeostasis with polyphenols and polyphenol-rich plant extracts from *in vitro* & animal studies.
- Epidemiological evidence supports beneficial effects of polyphenol- rich diets.
- Clinical studies so far have not undoubtedly succeeded in pointing out any specific polyphenols or food products in reducing the risk of insulin resistance.
- It is evident that in clinical studies whole diets instead of single compounds or food components should be addressed.
- Combination of specific clinical measurements determining glucose tolerance and insulin sensitivity together with systems biology profiling technologies is needed to get a holistic view on the health effects of diets and foods rich in polyphenols.

## Figures and Tables

**Table 1.** Effect of polyphenols on carbohydrate homeostasis measured *in vitro*.

	**Inhibiotion of α–Amylase activity**	**Inhibiotion of α–Glucosidase activity**	**Inhibition of glucose absorption from intestine**	**Protection of β-cells in cell culture**	**Protection of β-cells in islets or pancreas**	**Increased insulin secretion/content from cultured cells**	**Increased insulin secretion/content in isolated islets/pancreas**	**Improved GU in muscle cells**	**Improved GU in adypocytes**	**Induction of hepatic glucokinase activity**	**Supression of gluconeogenetic enzyme expression**	**Activation of hepatic AMPK**
***Anthocyanins***												
Cyanidin 3-galactoside		[191]										
Cyanidin 3-rutinoside		[192]										
Cyanidin 3-sambubioside	[183]	[183]										
Cyanidin 3-glucoside						[70]						
Acylated anthocyanins		[193,194]										
Delphinidin 3-glucoside						[70]						
***Catechins***												
Catechin		[43]	[33,37]									
Epicatechin					[78]		[78,79]					
Catechin gallate		[43]										
Epi(gallo)catechin		[33,43,195]	[37]									
Epi(gallo)catechin gallate		[41,184]	[35–37]	[65]		[65]		[95]		[135]	[135–137]	[136,138,151]
Theaflavin												[151]
Theaflavin gallate		[41]										[151]
Theaflavin digallate												[151]
***Flavonols, flavones, flavanones***		[184]										
Naringenin			[38]									
Naringin										[143,144]	[143,144]	
Quercetin	[184,185]	[43,184]	[37,39]	[73]	[76,77]		[73,76,79]		[101]			
Quercetin 3-glucoside			[34]									
Quercetin 4’-glucoside			[34]									
Quercetin 3-rhamnoside		[196]										
Rutin				[65, 74]		[65]	[85]					
Myricetin	[184,185]	[184]	[37]									
Hesperidin										[143,144]	[144]	
Apigenin			[37]	[73]			[73,85]					
Luteolin	[184–187]	[184,186]		[73]			[73]					
Luteolin 7-glucoside	[186]	[186]										
Puerarin								[205]				
Kaempferol									[101]			
Kaempferol 3-neohesperidoside								[102]				
***Isoflavones***												
Genistein		[184,197]				[69,201,202]	[62,69,202–204]			[139]	[62,139]	
Daidzein		[184,186]								[62,139]	[62,139]	
3,5-Diprenylgenistein								[120]				
6,8-Diprenylgenistein								[120]				
Alpinumisoflavone								[120]				
Derrone								[120]				
***Phenolic acids***												
Caffeic acid	[188]	[43,195,198]	[33]									
Chlorogenic acid	[187,188]	[43,183]	[33,43]					[94]				
*p*-Coumaric acid		[195]										
Ferulic acid		[195, 198]	[33]			[67]	[67]	[94]		[150]		
Gallic acid		[195]										
Tannic acid	[187]	[195,199]	[33]									
***Ellagitannins***	[189]											
***Proanthocyanidins***	[190]	[190, 200]										
***Other phenolics***												
Aspalathin						[71]		[71]				
Penta-galloyl-glucose									[206]			
Resveratrol								[96,98]				[154]

**Table 2.** Effect of dietary plants or extracts on carbohydrate homeostasis measured *in vitro*.

	**Inhibiotion of a–Amylase activity**	**Inhibiotion of a–Glucosidase activity**	**Inhibition of glucose absorption from intestine**	**Protection of beta-cells in cell culture**	**Protection of beta-cells in islets or pancreas**	**Increased insulin secretion/content from cultured cells**	**Increased insulin secretion/content in isolated islets/pancreas**	**Improved GU in muscle cells**	**Improved GU in adypocytes**	**Induction of hepatic glucokinase activity**	**Supression of gluconeogenetic enzyme expression**	**Activation of hepatic AMPK**
Acerola		[196]	[42]									
Berries; strawberry, raspberry, blueberry, blackcurrant	[189]	[189,213]										
Blueberry				[72]		[72]		[114]	[114]			
Black rice		[214]										
Rice bran										[150]		
*Gingko biloba*	[44,207]	[44,207]										
Red wine	[189]	[215]										
Tea green, black	[189,208, 209,210]	[209,215]							[181]	[218]	[218]	
Vegetables; pumpkin, beans, maize, eggplant	[211,212]	[211,212]										
Soy					[64]		[63,64,216]			[63]	[63]	[140]
Grape					[80]							
Grape seed								[108, 109]	[108]	[173]		
Bitter melon								[110]	[111]			
*Canna indica* root								[122]				
Cinnamon									[217]			
*Artemisia dracunculus*										[219]		

## References

[1] Ovaskainen ML Torronen R Koponen JM Sinkko H Hellstrom J Reinivuo H Mattila P Dietary intake and major food sources of polyphenols in Finnish adults J. Nutr 2008 138 562 566 18287367 10.1093/jn/138.3.562

[2] Scalbert A Manach C Morand C Remesy C Jimenez L Dietary polyphenols and the prevention of diseases Crit. Rev. Food Sci. Nutr 2005 45 287 306 16047496 10.1080/1040869059096

[3] Crozier A Jaganath IB Clifford MN Dietary phenolics: Chemistry, bioavailability and effects on health Nat. Prod. Rep 2009 26 1001 1043 19636448 10.1039/b802662a

[4] Clifford MN Diet-derived phenols in plasma and tissues and their implications for health Planta Med 2004 70 1103 1114 15643541 10.1055/s-2004-835835

[5] Knekt P Kumpulainen J Jarvinen R Rissanen H Heliovaara M Reunanen A Hakulinen T Aromaa A Flavonoid intake and risk of chronic diseases Am. J. Clin. Nutr 2002 76 560 568 12198000 10.1093/ajcn/76.3.560

[6] Selma MV Espin JC Tomas-Barberan FA Interaction between phenolics and gut microbiota: role in human health J. Agric. Food Chem 2009 57 6485 6501 19580283 10.1021/jf902107d

[7] Lila MA From beans to berries and beyond: Teamwork between plant chemicals for protection of optimal human health Ann. NY Acad. Sci 2007 1114 372 380 17986598 10.1196/annals.1396.047

[8] Liu RH Health benefits of fruit and vegetables are from additive and synergistic combinations of phytochemicals Am. J. Clin. Nutr 2003 78 517S 520S 12936943 10.1093/ajcn/78.3.517S

[9] Eckel RH Grundy SM Zimmet PZ The metabolic syndrome Lancet 2005 365 1415 1428 15836891 10.1016/S0140-6736(05)66378-7

[10] Uusitupa M Gene-diet interaction in relation to the prevention of obesity and type 2 diabetes: Evidence from the Finnish Diabetes Prevention Study Nutr. Metab. Cardiovasc. Dis 2005 15 225 233 15955472 10.1016/j.numecd.2005.03.004

[11] McCarthy MI Progress in defining the molecular basis of type 2 diabetes mellitus through susceptibility-gene identification Hum Mol Genet 2004 13 Spec No 1 R33 R41 14722160 10.1093/hmg/ddh057

[12] Laaksonen DE Niskanen L Lakka HM Lakka TA Uusitupa M Epidemiology and treatment of the metabolic syndrome Ann. Med 2004 36 332 346 15478308 10.1080/07853890410031849

[13] Lann D Gallagher E Leroith D Insulin resistance and the metabolic syndrome Minerva Med 2008 99 253 262 18497723

[14] Wild S Roglic G Green A Sicree R King H Global prevalence of diabetes: Estimates for the year 2000 and projections for 2030 Diabetes Care 2004 27 1047 1053 15111519 10.2337/diacare.27.5.1047

[15] Tuomilehto J Lindstrom J Eriksson JG Valle TT Hamalainen H Ilanne-Parikka P Keinanen-Kiukaanniemi S Laakso M Louheranta A Rastas M Salminen V Uusitupa M Finnish Diabetes Prevention Study Group Prevention of type 2 diabetes mellitus by changes in lifestyle among subjects with impaired glucose tolerance N. Engl. J. Med 2001 344 1343 1350 11333990 10.1056/NEJM200105033441801

[16] Lindström J Ilanne-Parikka P Peltonen M Aunola S Eriksson JG Hemiö K Hämäläinen H Härkönen P Keinänen-Kiukaanniemi S Laakso M Louheranta A Mannelin M Paturi M Sundvall J Valle TT Uusitupa M Tuomilehto J Sustained reduction in the incidence of type 2 diabetes by lifestyle intervention: Follow-up of the Finnish Diabetes Prevention Study Lancet 2006 368 1673 1679 17098085 10.1016/S0140-6736(06)69701-8

[17] Orgaard A Jensen L The effects of soy isoflavones on obesity. *Exp. Biol. Med.* (Maywood) 2008 233 1066 1080 10.3181/0712-MR-347 18535167

[18] Wolfram S Effects of green tea and EGCG on cardiovascular and metabolic health J. Am. Coll. Nutr 2007 26 373S 388S 17906191 10.1080/07315724.2007.10719626

[19] van Dam RM Hu FB Coffee consumption and risk of type 2 diabetes: A systematic review JAMA 2005 294 97 104 15998896 10.1001/jama.294.1.97

[20] Zunino S Type 2 diabetes and glycemic response to grapes or grape products J. Nutr 2009 139 1794S 800S 19625702 10.3945/jn.109.107631

[21] Boyer J Liu RH Apple phytochemicals and their health benefits Nutr. J 2004 3 5 15140261 10.1186/1475-2891-3-5 PMC442131

[22] Hui H Tang G Go VL Hypoglycemic herbs and their action mechanisms Chin. Med 2009 4 11 19523223 10.1186/1749-8546-4-11 PMC2704217

[23] Ghosh D Konishi T Anthocyanins and anthocyanin-rich extracts: Role in diabetes and eye function Asia Pac. J. Clin. Nutr 2007 16 200 208 17468073

[24] Bondia-Pons I Aura A Vuorela S Kolehmainen M Mykkanen H Poutanen K Rye phenolics in nutrition and health J. Cereal Sci 2009 49 323 336

[25] Maritim AC Sanders RA Watkins JB III Diabetes, oxidative stress, and antioxidants: A review J. Biochem. Mol. Toxicol 2003 17 24 38 12616644 10.1002/jbt.10058

[26] Vincent HK Innes KE Vincent KR Oxidative stress and potential interventions to reduce oxidative stress in overweight and obesity Diabetes Obes. Metab 2007 9 813 839 17924865 10.1111/j.1463-1326.2007.00692.x

[27] Ludwig DS The glycemic index: Physiological mechanisms relating to obesity, diabetes, and cardiovascular disease JAMA 2002 287 2414 2423 11988062 10.1001/jama.287.18.2414

[28] Quezada-Calvillo R Robayo-Torres CC Ao Z Hamaker BR Quaroni A Brayer GD Sterchi EE Baker SS Nichols BL Luminal substrate “brake” on mucosal maltase-glucoamylase activity regulates total rate of starch digestion to glucose J. Pediatr. Gastroenterol. Nutr 2007 45 32 43 17592362 10.1097/MPG.0b013e31804216fc

[29] Quezada-Calvillo R Robayo-Torres CC Opekun AR Sen P Ao Z Hamaker BR Quaroni A Brayer GD Wattler S Nehls MC Sterchi EE Nichols BL Contribution of mucosal maltase-glucoamylase activities to mouse small intestinal starch alpha-glucogenesis J. Nutr 2007 137 1725 1733 17585022 10.1093/jn/137.7.1725

[30] Quezada-Calvillo R Sim L Ao Z Hamaker BR Quaroni A Brayer GD Sterchi EE Robayo-Torres CC Rose DR Nichols BL Luminal starch substrate “brake” on maltase-glucoamylase activity is located within the glucoamylase subunit J. Nutr 2008 138 685 692 18356321 10.1093/jn/138.4.685

[31] Levin RJ Digestion and absorption of carbohydrates--from molecules and membranes to humans Am. J. Clin. Nutr 1994 59 690S 698S 8116552 10.1093/ajcn/59.3.690S

[32] Drozdowski LA Thomson ABR Intestinal sugar transport World J. Gastroenterol 2006 12 1657 1670 16586532 10.3748/wjg.v12.i11.1657 PMC4124338

[33] Welsch CA Lachance PA Wasserman BP Dietary phenolic compounds: Inhibition of Na^+^- dependent D-glucose uptake in rat intestinal brush border membrane vesicles J. Nutr 1989 119 1698 1704 2600675 10.1093/jn/119.11.1698

[34] Cermak R Landgraf S Wolffram S Quercetin glucosides inhibit glucose uptake into brush-border-membrane vesicles of porcine jejunum Br. J. Nutr 2004 91 849 855 15182388 10.1079/BJN20041128

[35] Kobayashi Y Suzuki M Satsu H Arai S Hara Y Suzuki K Miyamoto Y Shimizu M Green tea polyphenols inhibit the sodium-dependent glucose transporter of intestinal epithelial cells by a competitive mechanism J. Agric. Food Chem 2000 48 5618 5623 11087528 10.1021/jf0006832

[36] Shimizu M Kobayashi Y Suzuki M Satsu H Miyamoto Y Regulation of intestinal glucose transport by tea catechins Biofactors 2000 13 61 65 11237201 10.1002/biof.5520130111

[37] Johnston K Sharp P Clifford M Morgan L Dietary polyphenols decrease glucose uptake by human intestinal Caco-2 cells FEBS Lett 2005 579 1653 1657 15757656 10.1016/j.febslet.2004.12.099

[38] Li JM Che CT Lau CB Leung PS Cheng CH Inhibition of intestinal and renal Na^+^- glucose cotransporter by naringenin Int. J. Biochem. Cell Biol 2006 38 985 995 16289850 10.1016/j.biocel.2005.10.002

[39] Song J Kwon O Chen S Daruwala R Eck P Park JB Levine M Flavonoid inhibition of sodium-dependent vitamin C transporter 1 (SVCT1) and glucose transporter isoform 2 (GLUT2), intestinal transporters for vitamin C and Glucose J. Biol. Chem 2002 277 15252 15260 11834736 10.1074/jbc.M110496200

[40] Matsui T Ebuchi S Kobayashi M Fukui K Sugita K Terahara N Matsumoto K Antihyperglycemic effect of diacylated anthocyanin derived from Ipomoea batatas cultivar Ayamurasaki can be achieved through the alpha-glucosidase inhibitory action J. Agric. Food Chem 2002 50 7244 7248 12452639 10.1021/jf025913m

[41] Matsui T Tanaka T Tamura S Toshima A Tamaya K Miyata Y Tanaka K Matsumoto K alpha-Glucosidase inhibitory profile of catechins and theaflavins J. Agric. Food Chem 2007 55 99 105 17199319 10.1021/jf0627672

[42] Hanamura T Mayama C Aoki H Hirayama Y Shimizu M Antihyperglycemic effect of polyphenols from Acerola (Malpighia emarginata DC.) fruit Biosci. Biotechnol. Biochem 2006 70 1813 1820 16926491 10.1271/bbb.50592

[43] Ishikawa A Yamashita H Hiemori M Inagaki E Kimoto M Okamoto M Tsuji H Memon AN Mohammadio A Natori Y Characterization of inhibitors of postprandial hyperglycemia from the leaves of Nerium indicum J. Nutr. Sci. Vitaminol. (Tokyo) 2007 53 166 173 17616005 10.3177/jnsv.53.166

[44] Tanaka S Han LK Zheng YN Okuda H Effects of the flavonoid fraction from Ginkgo biloba extract on the postprandial blood glucose elevation in rats Yakugaku Zasshi 2004 124 605 611 15340182 10.1248/yakushi.124.605

[45] Johnston KL Clifford MN Morgan LM Possible role for apple juice phenolic, compounds in the acute modification of glucose tolerance and gastrointestinal hormone secretion in humans J. Sci. Food Agric 2002 82 1800 1805

[46] Torronen R Sarkkinen E Tapola N Hautaniemi E Kilpi K Niskanen L Berries modify the postprandial plasma glucose response to sucrose in healthy subjects Br J Nutr 2009E-pub ahead of a print 10.1017/S0007114509992868 19930765

[47] Wilson T Singh AP Vorsa N Goettl CD Kittleson KM Roe CM Kastello GM Ragsdale FR Human glycemic response and phenolic content of unsweetened cranberry juice J. Med. Food 2008 11 46 54 18361737 10.1089/jmf.2007.531

[48] Gin H Rigalleau V Caubet O Masquelier J Aubertin J Effects of red wine, tannic acid, or ethanol on glucose tolerance in non-insulin-dependent diabetic patients and on starch digestibility *in vitro.* Metabolism 1999 48 1179 1183 10484061 10.1016/s0026-0495(99)90135-x

[49] Holt S Jong VD Faramus E Lang T Brand Miller J A bioflavonoid in sugar cane can reduce the postprandial glycaemic response to a high-GI starchy food Asia Pac. J. Clin. Nutr 2003 12 S6615023698

[50] Hlebowicz J Darwiche G Bjorgell O Almer LO Effect of cinnamon on postprandial blood glucose, gastric emptying, and satiety in healthy subjects Am. J. Clin. Nutr 2007 85 1552 1556 17556692 10.1093/ajcn/85.6.1552

[51] Hlebowicz J Hlebowicz A Lindstedt S Bjorgell O Hoglund P Holst JJ Darwiche G Almer LO Effects of 1 and 3 g cinnamon on gastric emptying, satiety, and postprandial blood glucose, insulin, glucose-dependent insulinotropic polypeptide, glucagon-like peptide 1, and ghrelin concentrations in healthy subjects Am. J. Clin. Nutr 2009 89 815 821 19158209 10.3945/ajcn.2008.26807

[52] Johnston KL Clifford MN Morgan LM Coffee acutely modifies gastrointestinal hormone secretion and glucose tolerance in humans: Glycemic effects of chlorogenic acid and caffeine Am. J. Clin. Nutr 2003 78 728 733 14522730 10.1093/ajcn/78.4.728

[53] van Dijk AE Olthof MR Meeuse JC Seebus E Heine RJ van Dam RM Acute effects of decaffeinated coffee and the major coffee components chlorogenic acid and trigonelline on glucose tolerance Diabetes Care 2009 32 1023 1025 19324944 10.2337/dc09-0207 PMC2681030

[54] Thom E The effect of chlorogenic acid enriched coffee on glucose absorption in healthy volunteers and its effect on body mass when used long-term in overweight and obese people J. Int. Med. Res 2007 35 900 908 18035001 10.1177/147323000703500620

[55] Aldughpassi A Wolever TM Effect of coffee and tea on the glycaemic index of foods: No effect on mean but reduced variability Br. J. Nutr 2009 101 1282 1285 19434800 10.1017/s0007114508079610

[56] Battram DS Arthur R Weekes A Graham TE The glucose intolerance induced by caffeinated coffee ingestion is less pronounced than that due to alkaloid caffeine in men J. Nutr 2006 136 1276 1280 16614416 10.1093/jn/136.5.1276

[57] Moisey LL Kacker S Bickerton AC Robinson LE Graham TE Caffeinated coffee consumption impairs blood glucose homeostasis in response to high and low glycemic index meals in healthy men Am. J. Clin. Nutr 2008 87 1254 1261 18469247 10.1093/ajcn/87.5.1254

[58] Bryans JA Judd PA Ellis PR The effect of consuming instant black tea on postprandial plasma glucose and insulin concentrations in healthy humans J. Am. Coll. Nutr 2007 26 471 477 17914136 10.1080/07315724.2007.10719638

[59] Rutter GA Nutrient-secretion coupling in the pancreatic islet beta-cell: Recent advances Mol. Aspects Med 2001 22 247 284 11890977 10.1016/s0098-2997(01)00013-9

[60] Rutter GA Visualising insulin secretion. The Minkowski Lecture 2004 Diabetologia 2004 47 1861 1872 15551048 10.1007/s00125-004-1541-1

[61] Chang-Chen KJ Mullur R Bernal-Mizrachi E Beta-cell failure as a complication of diabetes Rev. Endocr Metab. Disord 2008 9 329 343 18777097 10.1007/s11154-008-9101-5 PMC4456188

[62] Choi MS Jung UJ Yeo J Kim MJ Lee MK Genistein and daidzein prevent diabetes onset by elevating insulin level and altering hepatic gluconeogenic and lipogenic enzyme activities in non-obese diabetic (NOD) mice Diabetes Metab. Res. Rev 2008 24 74 81 17932873 10.1002/dmrr.780

[63] Kim DJ Jeong YJ Kwon JH Moon KD Kim HJ Jeon SM Lee MK Park YB Choi MS Beneficial effect of chungkukjang on regulating blood glucose and pancreatic beta-cell functions in C75BL/KsJ-db/db mice J. Med. Food 2008 11 215 223 18598161 10.1089/jmf.2007.560

[64] Lu MP Wang R Song X Chibbar R Wang X Wu L Meng QH Dietary soy isoflavones increase insulin secretion and prevent the development of diabetic cataracts in streptozotocin-induced diabetic rats Nutr. Res 2008 28 464 471 19083447 10.1016/j.nutres.2008.03.009

[65] Cai EP Lin JK Epigallocatechin Gallate (EGCG) and rutin suppress the glucotoxicity through activating IRS2 and AMPK signaling in rat pancreatic beta cells J Agric Food Chem 2009 10 5 [Epub ahead of print] 10.1021/jf902618v 19803520

[66] Qa’dan F Verspohl EJ Nahrstedt A Petereit F Matalka KZ Cinchonain Ib isolated from Eriobotrya japonica induces insulin secretion *in vitro* and *in vivo* J. Ethnopharmacol 2009 124 224 227 19397981 10.1016/j.jep.2009.04.023

[67] Adisakwattana S Moonsan P Yibchok-Anun S Insulin-releasing properties of a series of cinnamic acid derivatives *in vitro* and *in vivo* J. Agric. Food Chem 2008 56 7838 7844 18651742 10.1021/jf801208t

[68] Liu IM Chen WC Cheng JT Mediation of beta-endorphin by isoferulic acid to lower plasma glucose in streptozotocin-induced diabetic rats J. Pharmacol. Exp. Ther 2003 307 1196 1204 12975496 10.1124/jpet.103.053900

[69] Fu Z Liu D Long-term exposure to genistein improves insulin secretory function of pancreatic beta-cells Eur. J. Pharmacol 2009 616 321 327 19540219 10.1016/j.ejphar.2009.06.005 PMC2720420

[70] Jayaprakasam B Vareed SK Olson LK Nair MG Insulin secretion by bioactive anthocyanins and anthocyanidins present in fruits J. Agric. Food Chem 2005 53 28 31 15631504 10.1021/jf049018+

[71] Kawano A Nakamura H Hata S Minakawa M Miura Y Yagasaki K Hypoglycemic effect of aspalathin, a rooibos tea component from Aspalathus linearis, in type 2 diabetic model db/db mice Phytomedicine 2009 16 437 443 19188054 10.1016/j.phymed.2008.11.009

[72] Martineau LC Couture A Spoor D Benhaddou-Andaloussi A Harris C Meddah B Leduc C Burt A Vuong T Mai Le P Prentki M Bennett SA Arnason JT Haddad PS Anti-diabetic properties of the Canadian lowbush blueberry Vaccinium angustifolium Ait Phytomedicine 2006 13 612 623 16979328 10.1016/j.phymed.2006.08.005

[73] Kim EK Kwon KB Song MY Han MJ Lee JH Lee YR Lee JH Ryu DG Park BH Park JW Flavonoids protect against cytokine-induced pancreatic beta-cell damage through suppression of nuclear factor kappaB activation Pancreas 2007 35 e1 e9 18090225 10.1097/mpa.0b013e31811ed0d2

[74] Stanley Mainzen Prince P Kamalakkannan N Rutin improves glucose homeostasis in streptozotocin diabetic tissues by altering glycolytic and gluconeogenic enzymes J. Biochem. Mol. Toxicol 2006 20 96 102 16615078 10.1002/jbt.20117

[75] Kobori M Masumoto S Akimoto Y Takahashi Y Dietary quercetin alleviates diabetic symptoms and reduces streptozotocin-induced disturbance of hepatic gene expression in mice Mol. Nutr. Food Res 2009 53 859 868 19496084 10.1002/mnfr.200800310

[76] Coskun O Kanter M Korkmaz A Oter S Quercetin, a flavonoid antioxidant, prevents and protects streptozotocin-induced oxidative stress and beta-cell damage in rat pancreas Pharmacol. Res 2005 51 117 123 15629256 10.1016/j.phrs.2004.06.002

[77] Vessal M Hemmati M Vasei M Antidiabetic effects of quercetin in streptozocin-induced diabetic rats Comp. Biochem. Physiol. C. Toxicol. Pharmacol 2003 135C 357 364 12927910 10.1016/s1532-0456(03)00140-6

[78] Chakravarthy BK Gupta S Gode KD Functional beta cell regeneration in the islets of pancreas in alloxan induced diabetic rats by (−)-epicatechin Life Sci 1982 31 2693 2697 6759833 10.1016/0024-3205(82)90713-5

[79] Hii CS Howell SL Effects of flavonoids on insulin secretion and 45Ca^2+^ handling in rat islets of Langerhans J. Endocrinol 1985 107 1 8 3900267 10.1677/joe.0.1070001

[80] Zunino SJ Storms DH Stephensen CB Diets rich in polyphenols and vitamin A inhibit the development of type I autoimmune diabetes in nonobese diabetic mice J. Nutr 2007 137 1216 1221 17449584 10.1093/jn/137.5.1216

[81] Hamden K Allouche N Damak M Elfeki A Hypoglycemic and antioxidant effects of phenolic extracts and purified hydroxytyrosol from olive mill waste *in vitro* and in rats Chem. Biol. Interact 2009 180 421 432 19393637 10.1016/j.cbi.2009.04.002

[82] Sharma B Balomajumder C Roy P Hypoglycemic and hypolipidemic effects of flavonoid rich extract from Eugenia jambolana seeds on streptozotocin induced diabetic rats Food Chem. Toxicol 2008 46 2376 2383 18474411 10.1016/j.fct.2008.03.020

[83] Kang YJ Jung UJ Lee MK Kim HJ Jeon SM Park YB Chung HG Baek NI Lee KT Jeong TS Choi MS Eupatilin, isolated from Artemisia princeps Pampanini, enhances hepatic glucose metabolism and pancreatic beta-cell function in type 2 diabetic mice Diabetes Res. Clin. Pract 2008 82 25 32 18703253 10.1016/j.diabres.2008.06.012

[84] Krisanapun C Peungvicha P Temsiririrkkul R Wongkrajang Y Aqueous extract of Abutilon indicum Sweet inhibits glucose absorption and stimulates insulin secretion in rodents Nutr. Res 2009 29 579 587 19761892 10.1016/j.nutres.2009.07.006

[85] Esmaeili MA Zohari F Sadeghi H Antioxidant and protective effects of major flavonoids from Teucrium polium on beta-cell destruction in a model of streptozotocin-induced diabetes Planta Med 2009 75 1418 1420 19452438 10.1055/s-0029-1185704

[86] Zaid H Antonescu CN Randhawa VK Klip A Insulin action on glucose transporters through molecular switches, tracks and tethers Biochem. J 2008 413 201 215 18570632 10.1042/BJ20080723

[87] Bjornholm M Zierath JR Insulin signal transduction in human skeletal muscle: Identifying the defects in Type II diabetes Biochem. Soc. Trans 2005 33 354 357 15787605 10.1042/BST0330354

[88] Uldry M Thorens B The SLC2 family of facilitated hexose and polyol transporters Pflugers Arch 2004 447 480 489 12750891 10.1007/s00424-003-1085-0

[89] Konrad D Somwar R Sweeney G Yaworsky K Hayashi M Ramlal T Klip A The antihyperglycemic drug alpha-lipoic acid stimulates glucose uptake *via* both GLUT4 translocation and GLUT4 activation: Potential role of p38 mitogen-activated protein kinase in GLUT4 activation Diabetes 2001 50 1464 1471 11375349 10.2337/diabetes.50.6.1464

[90] Liu W Hsin C Tang F A molecular mathematical model of glucose mobilization and uptake Math. Biosci 2009 221 121 129 19651146 10.1016/j.mbs.2009.07.005

[91] Taniguchi CM Kondo T Sajan M Luo J Bronson R Asano T Farese R Cantley LC Kahn CR Divergent regulation of hepatic glucose and lipid metabolism by phosphoinositide 3-kinase *via* Akt and PKClambda/zeta Cell. Metab 2006 3 343 353 16679292 10.1016/j.cmet.2006.04.005

[92] Dugani CB Randhawa VK Cheng AW Patel N Klip A Selective regulation of the perinuclear distribution of glucose transporter 4 (GLUT4) by insulin signals in muscle cells Eur. J. Cell Biol 2008 87 337 351 18417252 10.1016/j.ejcb.2008.02.009

[93] Saltiel AR Kahn CR Insulin signalling and the regulation of glucose and lipid metabolism Nature 2001 414 799 806 11742412 10.1038/414799a

[94] Prabhakar PK Doble M Synergistic effect of phytochemicals in combination with hypoglycemic drugs on glucose uptake in myotubes Phytomedicine 2009 16 1119 1126 19660925 10.1016/j.phymed.2009.05.021

[95] Zhang ZF Li Q Liang J Dai XQ Ding Y Wang JB Li Y Epigallocatechin-3-O-gallate (EGCG) protects the insulin sensitivity in rat L6 muscle cells exposed to dexamethasone condition Phytomedicine 2010 17 14 18 19819682 10.1016/j.phymed.2009.09.007

[96] Park CE Kim MJ Lee JH Min BI Bae H Choe W Kim SS Ha J Resveratrol stimulates glucose transport in C2C12 myotubes by activating AMP-activated protein kinase Exp. Mol. Med 2007 39 222 229 17464184 10.1038/emm.2007.25

[97] Deng JY Hsieh PS Huang JP Lu LS Hung LM Activation of estrogen receptor is crucial for resveratrol-stimulating muscular glucose uptake *via* both insulin-dependent and - independent pathways Diabetes 2008 57 1814 1823 18426865 10.2337/db07-1750 PMC2453636

[98] Breen DM Sanli T Giacca A Tsiani E Stimulation of muscle cell glucose uptake by resveratrol through sirtuins and AMPK Biochem. Biophys. Res. Commun 2008 374 117 122 18601907 10.1016/j.bbrc.2008.06.104

[99] Lagouge M Argmann C Gerhart-Hines Z Meziane H Lerin C Daussin F Messadeq N Milne J Lambert P Elliott P Geny B Laakso M Puigserver P Auwerx J Resveratrol improves mitochondrial function and protects against metabolic disease by activating SIRT1 and PGC-1alpha Cell 2006 127 1109 1122 17112576 10.1016/j.cell.2006.11.013

[100] Milne JC Small molecule activators of SIRT1 as therapeutics for the treatment of type 2 diabetes Nature 2007 450 712 716 18046409 10.1038/nature06261 PMC2753457

[101] Fang XK Gao J Zhu DN Kaempferol and quercetin isolated from Euonymus alatus improve glucose uptake of 3T3-L1 cells without adipogenesis activity Life Sci 2008 82 615 622 18262572 10.1016/j.lfs.2007.12.021

[102] Zanatta L Rosso A Folador P Figueiredo MS Pizzolatti MG Leite LD Silva FR Insulinomimetic effect of kaempferol 3-neohesperidoside on the rat soleus muscle J. Nat. Prod 2008 71 532 535 18303854 10.1021/np070358+

[103] Vishnu Prasad CN Suma Mohan S Banerji A Gopalakrishnapillai A Kaempferitrin inhibits GLUT4 translocation and glucose uptake in 3T3-L1 adipocytes Biochem. Biophys. Res. Commun 2009 380 39 43 19146827 10.1016/j.bbrc.2009.01.008

[104] Tzeng YM Chen K Rao YK Lee MJ Kaempferitrin activates the insulin signaling pathway and stimulates secretion of adiponectin in 3T3-L1 adipocytes Eur. J. Pharmacol 2009 607 27 34 19326566 10.1016/j.ejphar.2009.01.023

[105] Jorge AP Horst H de Sousa E Pizzolatti MG Silva FR Insulinomimetic effects of kaempferitrin on glycaemia and on 14C-glucose uptake in rat soleus muscle Chem. Biol. Interact 2004 149 89 96 15501431 10.1016/j.cbi.2004.07.001

[106] Bazuine M van den Broek PJ Maassen JA Genistein directly inhibits GLUT4-mediated glucose uptake in 3T3-L1 adipocytes Biochem. Biophys. Res. Commun 2005 326 511 514 15582607 10.1016/j.bbrc.2004.11.055

[107] Cao H Hininger-Favier I Kelly MA Benaraba R Dawson HD Coves S Roussel AM Anderson RA Green tea polyphenol extract regulates the expression of genes involved in glucose uptake and insulin signaling in rats fed a high fructose diet J. Agric. Food Chem 2007 55 6372 6378 17616136 10.1021/jf070695o

[108] Pinent M Blay M Blade MC Salvado MJ Arola L Ardevol A Grape seed-derived procyanidins have an antihyperglycemic effect in streptozotocin-induced diabetic rats and insulinomimetic activity in insulin-sensitive cell lines Endocrinology 2004 145 4985 4990 15271880 10.1210/en.2004-0764

[109] Montagut G Onnockx S Vaque M Blade C Blay M Fernandez-Larrea J Pujadas G Salvado MJ Arola L Pirson I Ardevol A Pinent M Oligomers of grape-seed procyanidin extract activate the insulin receptor and key targets of the insulin signaling pathway differently from insulin J Nutr Biochem 2009doi:10.1016/j.jnutbio.2009.02.003 10.1016/j.jnutbio.2009.02.003 19443198

[110] Cummings E Hundal HS Wackerhage H Hope M Belle M Adeghate E Singh J Momordica charantia fruit juice stimulates glucose and amino acid uptakes in L6 myotubes Mol. Cell. Biochem 2004 261 99 104 15362491 10.1023/b:mcbi.0000028743.75669.ab

[111] Roffey BW Atwal AS Johns T Kubow S Water extracts from Momordica charantia increase glucose uptake and adiponectin secretion in 3T3-L1 adipose cells J. Ethnopharmacol 2007 112 77 84 17363205 10.1016/j.jep.2007.02.003

[112] Kumar R Balaji S Uma TS Sehgal PK Fruit extracts of Momordica charantia potentiate glucose uptake and up-regulate Glut-4, PPAR gamma and PI3K J. Ethnopharmacol 2009 126 533 537 19744549 10.1016/j.jep.2009.08.048

[113] Martin LJ Matar C Increase of antioxidant capacity of the lowbush blueberry (Vaccinium angustifolium) during fermentation by a novel bacterium from the fruit microflora J. Sci. Food Agric 2005 85 1477 1484

[114] Vuong T Martineau LC Ramassamy C Matar C Haddad PS Fermented Canadian lowbush blueberry juice stimulates glucose uptake and AMP-activated protein kinase in insulin-sensitive cultured muscle cells and adipocytes Can. J. Physiol. Pharmacol 2007 85 956 965 18066143 10.1139/Y07-090

[115] Anderson RA Broadhurst CL Polansky MM Schmidt WF Khan A Flanagan VP Schoene NW Graves DJ Isolation and characterization of polyphenol type-A polymers from cinnamon with insulin-like biological activity J. Agric. Food Chem 2004 52 65 70 14709014 10.1021/jf034916b

[116] Imparl-Radosevich J Deas S Polansky MM Baedke DA Ingebritsen TS Anderson RA Graves DJ Regulation of PTP-1 and insulin receptor kinase by fractions from cinnamon: Implications for cinnamon regulation of insulin signalling Horm. Res 1998 50 177 182 9762007 10.1159/000023270

[117] Cao H Polansky MM Anderson RA Cinnamon extract and polyphenols affect the expression of tristetraprolin, insulin receptor, and glucose transporter 4 in mouse 3T3-L1 adipocytes Arch. Biochem. Biophys 2007 459 214 222 17316549 10.1016/j.abb.2006.12.034

[118] Qin B Nagasaki M Ren M Bajotto G Oshida Y Sato Y Cinnamon extract (traditional herb) potentiates *in vivo* insulin-regulated glucose utilization *via* enhancing insulin signaling in rats Diabetes Res. Clin. Pract 2003 62 139 148 14625128 10.1016/s0168-8227(03)00173-6

[119] Qin B Nagasaki M Ren M Bajotto G Oshida Y Sato Y Cinnamon extract prevents the insulin resistance induced by a high-fructose diet Horm. Metab. Res 2004 36 119 125 15002064 10.1055/s-2004-814223

[120] Lee MS Kim CH Hoang DM Kim BY Sohn CB Kim MR Ahn JS Genistein-derivatives from Tetracera scandens stimulate glucose-uptake in L6 myotubes Biol. Pharm. Bull 2009 32 504 508 19252305 10.1248/bpb.32.504

[121] Li BG Hasselgren PO Fang CH Insulin-like growth factor-I inhibits dexamethasone-induced proteolysis in cultured L6 myotubes through PI3K/Akt/GSK-3beta and PI3K/Akt/mTOR-dependent mechanisms Int. J. Biochem. Cell Biol 2005 37 2207 2216 15927518 10.1016/j.biocel.2005.04.008

[122] Purintrapiban J Suttajit M Forsberg NE Differential activation of glucose transport in cultured muscle cells by polyphenolic compounds from Canna indica L. Root Biol. Pharm. Bull 2006 29 1995 1998 17015939 10.1248/bpb.29.1995

[123] Chang L Chiang SH Saltiel AR Insulin signaling and the regulation of glucose transport Mol. Med 2004 10 65 71 16307172 10.2119/2005-00029.Saltiel PMC1431367

[124] Cazarolli LH Zanatta L Alberton EH Figueiredo MS Folador P Damazio RG Pizzolatti MG Silva FR Flavonoids: Cellular and molecular mechanism of action in glucose homeostasis Mini Rev. Med. Chem 2008 8 1032 1038 18782055 10.2174/138955708785740580

[125] Cherrington AD Banting Lecture 1997. Control of glucose uptake and release by the liver *in vivo* Diabetes 1999 48 1198 1214 10331429 10.2337/diabetes.48.5.1198

[126] Pilkis SJ Claus TH Hepatic gluconeogenesis/glycolysis: Regulation and structure/function relationships of substrate cycle enzymes Annu. Rev. Nutr 1991 11 465 515 1892710 10.1146/annurev.nu.11.070191.002341

[127] Dentin R Liu Y Koo SH Hedrick S Vargas T Heredia J Yates J III Montminy M Insulin modulates gluconeogenesis by inhibition of the coactivator TORC2 Nature 2007 449 366 369 17805301 10.1038/nature06128

[128] Lam TK Pocai A Gutierrez-Juarez R Obici S Bryan J Aguilar-Bryan L Schwartz GJ Rossetti L Hypothalamic sensing of circulating fatty acids is required for glucose homeostasis Nat. Med 2005 11 320 327 15735652 10.1038/nm1201

[129] Pocai A Lam TK Gutierrez-Juarez R Obici S Schwartz GJ Bryan J Aguilar-Bryan L Rossetti L Hypothalamic K(ATP) channels control hepatic glucose production Nature 2005 434 1026 1031 15846348 10.1038/nature03439

[130] Postic C Girard J Contribution of de novo fatty acid synthesis to hepatic steatosis and insulin resistance: Lessons from genetically engineered mice J. Clin. Invest 2008 118 829 838 18317565 10.1172/JCI34275 PMC2254980

[131] Stewart LK Wang Z Ribnicky D Soileau JL Cefalu WT Gettys TW Failure of dietary quercetin to alter the temporal progression of insulin resistance among tissues of C57BL/6J mice during the development of diet-induced obesity Diabetologia 2009 52 514 523 19142628 10.1007/s00125-008-1252-0 PMC2758024

[132] Roghani M Baluchnejadmojarad T Hypoglycemic and hypolipidemic effect and antioxidant activity of chronic epigallocatechin-gallate in streptozotocin-diabetic rats Pathophysiology 2010 17 55 59 19682872 10.1016/j.pathophys.2009.07.004

[133] Shrestha S Ehlers SJ Lee JY Fernandez ML Koo SI Dietary green tea extract lowers plasma and hepatic triglycerides and decreases the expression of sterol regulatory element-binding protein-1c mRNA and its responsive genes in fructose-fed, ovariectomized rats J. Nutr 2009 139 640 645 19193814 10.3945/jn.108.103341 PMC2666357

[134] Bose M Lambert JD Ju J Reuhl KR Shapses SA Yang CS The major green tea polyphenol, (−)-epigallocatechin-3-gallate, inhibits obesity, metabolic syndrome, and fatty liver disease in high-fat-fed mice J. Nutr 2008 138 1677 1683 18716169 10.1093/jn/138.9.1677 PMC2586893

[135] Wolfram S Raederstorff D Preller M Wang Y Teixeira SR Riegger C Weber P Epigallocatechin gallate supplementation alleviates diabetes in rodents J. Nutr 2006 136 2512 2518 16988119 10.1093/jn/136.10.2512

[136] Collins QF Liu HY Pi J Liu Z Quon MJ Cao W Epigallocatechin-3-gallate (EGCG), a green tea polyphenol, suppresses hepatic gluconeogenesis through 5′-AMP-activated protein kinase J. Biol. Chem 2007 282 30143 30149 17724029 10.1074/jbc.M702390200 PMC2408735

[137] Waltner-Law ME Wang XL Law BK Hall RK Nawano M Granner DK Epigallocatechin gallate, a constituent of green tea, represses hepatic glucose production J. Biol. Chem 2002 277 34933 34940 12118006 10.1074/jbc.M204672200

[138] Lin CL Lin JK Epigallocatechin gallate (EGCG) attenuates high glucose-induced insulin signaling blockade in human hepG2 hepatoma cells Mol. Nutr. Food Res 2008 52 930 939 18496818 10.1002/mnfr.200700437

[139] Ae Park S Choi MS Cho SY Seo JS Jung UJ Kim MJ Sung MK Park YB Lee MK Genistein and daidzein modulate hepatic glucose and lipid regulating enzyme activities in C57BL/KsJ-db/db mice Life Sci 2006 79 1207 1213 16647724 10.1016/j.lfs.2006.03.022

[140] Cederroth CR Vinciguerra M Gjinovci A Kühne F Klein M Cederroth M Caille D Suter M Neumann D James RW Doerge DR Wallimann T Meda P Foti M Rohner-Jeanrenaud F Vassalli JD Nef S Dietary phytoestrogens activate AMP-activated protein kinase with improvement in lipid and glucose metabolism Diabetes 2008 57 1176 1185 18420492 10.2337/db07-0630

[141] Liang H Ward WF PGC-1alpha: A key regulator of energy metabolism Adv. Physiol. Educ 2006 30 145 151 17108241 10.1152/advan.00052.2006

[142] Kim JA Mechanisms underlying beneficial health effects of tea catechins to improve insulin resistance and endothelial dysfunction Endocr Metab. Immune Disord. Drug Targets 2008 8 82 88 18537694 10.2174/187153008784534349

[143] Jung UJ Lee MK Park YB Kang MA Choi MS Effect of citrus flavonoids on lipid metabolism and glucose-regulating enzyme mRNA levels in type-2 diabetic mice Int. J. Biochem. Cell Biol 2006 38 1134 1145 16427799 10.1016/j.biocel.2005.12.002

[144] Jung UJ Lee MK Jeong KS Choi MS The hypoglycemic effects of hesperidin and naringin are partly mediated by hepatic glucose-regulating enzymes in C57BL/KsJ-db/db mice J. Nutr 2004 134 2499 2503 15465737 10.1093/jn/134.10.2499

[145] Purushotham A Tian M Belury MA The citrus fruit flavonoid naringenin suppresses hepatic glucose production from Fao hepatoma cells Mol. Nutr. Food Res 2009 53 300 307 19035551 10.1002/mnfr.200700514

[146] Ganjam GK Dimova EY Unterman TG Kietzmann T FoxO1 and HNF-4 are involved in regulation of hepatic glucokinase gene expression by resveratrol J Biol Chem 2009 10.1074/jbc.M109.045260 PMC2781477 19740748

[147] Frescas D Valenti L Accili D Nuclear trapping of the forkhead transcription factor FoxO1 *via* Sirt-dependent deacetylation promotes expression of glucogenetic genes J. Biol. Chem 2005 280 20589 20595 15788402 10.1074/jbc.M412357200

[148] Motta MC Divecha N Lemieux M Kamel C Chen D Gu W Bultsma Y McBurney M Guarente L Mammalian SIRT1 represses forkhead transcription factors Cell 2004 116 551 563 14980222 10.1016/s0092-8674(04)00126-6

[149] Rodgers JT Puigserver P Fasting-dependent glucose and lipid metabolic response through hepatic sirtuin 1 Proc. Natl. Acad. Sci. USA 2007 104 12861 12866 17646659 10.1073/pnas.0702509104 PMC1937557

[150] Jung EH Kim SR Hwang IK Ha TY Hypoglycemic effects of a phenolic acid fraction of rice bran and ferulic acid in C57BL/KsJ-db/db mice J. Agric. Food Chem 2007 55 9800 9804 17973443 10.1021/jf0714463

[151] Lin CL Huang HC Lin JK Theaflavins attenuate hepatic lipid accumulation through activating AMPK in human HepG2 cells J. Lipid Res 2007 48 2334 2343 17720960 10.1194/jlr.M700128-JLR200

[152] Hwang JT Kwon DY Yoon SH AMP-activated protein kinase: A potential target for the diseases prevention by natural occurring polyphenols N. Biotechnol 2009 26 17 22 19818314 10.1016/j.nbt.2009.03.005

[153] Zang M Xu S Maitland-Toolan KA Zuccollo A Hou X Jiang B Wierzbicki M Verbeuren TJ Cohen RA Polyphenols stimulate AMP-activated protein kinase, lower lipids, and inhibit accelerated atherosclerosis in diabetic LDL receptor-deficient mice Diabetes 2006 55 2180 2191 16873680 10.2337/db05-1188

[154] Ajmo JM Liang X Rogers CQ Pennock B You M Resveratrol alleviates alcoholic fatty liver in mice Am. J. Physiol. Gastrointest. Liver Physiol 2008 295 G833 G842 18755807 10.1152/ajpgi.90358.2008 PMC2575919

[155] Cheng Z Guo S Copps K Dong X Kollipara R Rodgers JT Depinho RA Puigserver P White MF Foxo1 integrates insulin signaling with mitochondrial function in the liver Nat. Med 2009 15 1307 1311 19838201 10.1038/nm.2049 PMC3994712

[156] Song Y Manson JE Buring JE Sesso HD Liu S Associations of dietary flavonoids with risk of type 2 diabetes, and markers of insulin resistance and systemic inflammation in women: A prospective study and cross-sectional analysis J. Am. Coll. Nutr 2005 24 376 384 16192263 10.1080/07315724.2005.10719488

[157] Murtaugh MA Jacobs DR Jr Jacob B Steffen LM Marquart L Epidemiological support for the protection of whole grains against diabetes Proc. Nutr. Soc 2003 62 143 149 12740069 10.1079/pns2002223

[158] de Munter JS Hu FB Spiegelman D Franz M van Dam RM Whole grain, bran, and germ intake and risk of type 2 diabetes: A prospective cohort study and systematic review PLoS Med 2007 4 e261 17760498 10.1371/journal.pmed.0040261 PMC1952203

[159] Pereira MA Parker ED Folsom AR Coffee consumption and risk of type 2 diabetes mellitus: An 11-year prospective study of 28 812 postmenopausal women Arch. Int. Med 2006 166 1311 1316 16801515 10.1001/archinte.166.12.1311

[160] Jing Y Han G Hu Y Bi Y Li L Zhu D Tea consumption and risk of type 2 diabetes: A meta-analysis of cohort studies J. Gen. Intern. Med 2009 24 557 562 19308337 10.1007/s11606-009-0929-5 PMC2669862

[161] Polychronopoulos E Zeimbekis A Kastorini CM Papairakleous N Vlachou I Bountziouka V Panagiotakos DB Effects of black and green tea consumption on blood glucose levels in non-obese elderly men and women from Mediterranean Islands (MEDIS epidemiological study) Eur. J. Nutr 2008 47 10 16 18204918 10.1007/s00394-007-0690-7

[162] van Dieren S Uiterwaal CS van der Schouw YT van der A DL Boer JM Spijkerman A Grobbee DE Beulens JW Coffee and tea consumption and risk of type 2 diabetes Diabetologia 2009 52 2561 2569 19727658 10.1007/s00125-009-1516-3

[163] Brown AL Lane J Coverly J Stocks J Jackson S Stephen A Bluck L Coward A Hendrickx H Effects of dietary supplementation with the green tea polyphenol epigallocatechin-3-gallate on insulin resistance and associated metabolic risk factors: Randomized controlled trial Br. J. Nutr 2009 101 886 894 18710606 10.1017/S0007114508047727 PMC2819662

[164] Fukino Y Shimbo M Aoki N Okubo T Iso H Randomized controlled trial for an effect of green tea consumption on insulin resistance and inflammation markers J. Nutr. Sci. Vitaminol. (Tokyo) 2005 51 335 342 16392704 10.3177/jnsv.51.335

[165] Nagao T Meguro S Hase T Otsuka K Komikado M Tokimitsu I Yamamoto T Yamamoto K A catechin-rich beverage improves obesity and blood glucose control in patients with type 2 diabetes Obesity (Silver Spring) 2009 17 310 317 19008868 10.1038/oby.2008.505

[166] Grassi D Lippi C Necozione S Desideri G Ferri C Short-term administration of dark chocolate is followed by a significant increase in insulin sensitivity and a decrease in blood pressure in healthy persons Am. J. Clin. Nutr 2005 81 611 614 15755830 10.1093/ajcn/81.3.611

[167] Grassi D Desideri G Necozione S Lippi C Casale R Properzi G Blumberg JB Ferri C Blood pressure is reduced and insulin sensitivity increased in glucose-intolerant, hypertensive subjects after 15 days of consuming high-polyphenol dark chocolate J. Nutr 2008 138 1671 1676 18716168 10.1093/jn/138.9.1671

[168] Muniyappa R Hall G Kolodziej TL Karne RJ Crandon SK Quon MJ Cocoa consumption for 2 wk enhances insulin-mediated vasodilatation without improving blood pressure or insulin resistance in essential hypertension Am. J. Clin. Nutr 2008 88 1685 1696 19064532 10.3945/ajcn.2008.26457 PMC2969165

[169] Kar P Laight D Rooprai HK Shaw KM Cummings M Effects of grape seed extract in Type 2 diabetic subjects at high cardiovascular risk: A double blind randomized placebo controlled trial examining metabolic markers, vascular tone, inflammation, oxidative stress and insulin sensitivity Diabet. Med 2009 26 526 531 19646193 10.1111/j.1464-5491.2009.02727.x

[170] Andersen G Koehler P Somoza V Postprandial glucose and free fatty acid response is improved by wheat bread fortified with germinated wheat seedlings Curr. Topics Nutraceut. Res 2008 6 15 21

[171] Nahas R Moher M Complementary and alternative medicine for the treatment of type 2 diabetes Can. Fam. Physician 2009 55 591 596 19509199 PMC2694078

[172] Unno K Yamamoto H Maeda K Takabayashi F Yoshida H Kikunaga N Takamori N Asahina S Iguchi K Sayama K Hoshino M Protection of brain and pancreas from high-fat diet: Effects of catechin and caffeine Physiol. Behav 2009 96 262 269 18976677 10.1016/j.physbeh.2008.10.009

[173] Zhang HJ Ji BP Chen G Zhou F Luo YC Yu HQ Gao FY Zhang ZP Li HY A combination of grape seed-derived procyanidins and gypenosides alleviates insulin resistance in mice and HepG2 cells J. Food Sci 2009 74 H1 H7 19200096 10.1111/j.1750-3841.2008.00976.x

[174] DeFuria J Bennett G Strissel KJ Perfield JW II Milbury PE Greenberg AS Obin MS Dietary blueberry attenuates whole-body insulin resistance in high fat-fed mice by reducing adipocyte death and its inflammatory sequelae J. Nutr 2009 139 1510 1516 19515743 10.3945/jn.109.105155 PMC2709302

[175] Noriega-Lopez L Tovar AR Gonzalez-Granillo M Hernandez-Pando R Escalante B Santillan-Doherty P Torres N Pancreatic insulin secretion in rats fed a soy protein high fat diet depends on the interaction between the amino acid pattern and isoflavones J. Biol. Chem 2007 282 20657 20666 17507381 10.1074/jbc.M701045200

[176] Rivera L Moron R Sanchez M Zarzuelo A Galisteo M Quercetin ameliorates metabolic syndrome and improves the inflammatory status in obese Zucker rats Obesity (Silver Spring) 2008 16 2081 2087 18551111 10.1038/oby.2008.315

[177] Seymour M Tanone I Lewis S Urcuyo-Llanes D Bolling SF Bennink MR Blueberry-enriched diets reduce metabolic syndrome and insulin resistance in rats FASEB J 2009 23Available at: http://www.fasebj.org/cgi/content/meeting_abstract/23/1_MeetingAbstracts/563.31 (Accessed on 26 March 2010).

[178] Liu IM Tzeng TF Liou SS Lan TW Myricetin, a naturally occurring flavonol, ameliorates insulin resistance induced by a high-fructose diet in rats Life Sci 2007 81 1479 1488 17976658 10.1016/j.lfs.2007.08.045

[179] Kannappan S Anuradha CV Insulin sensitizing actions of fenugreek seed polyphenols, quercetin & metformin in a rat model Indian J. Med. Res 2009 129 401 408 19535835

[180] Tsai HY Wu LY Hwang LS Effect of a proanthocyanidin-rich extract from longan flower on markers of metabolic syndrome in fructose-fed rats J. Agric. Food Chem 2008 56 11018 11024 18973337 10.1021/jf801966y

[181] Wu LY Juan CC Ho LT Hsu YP Hwang LS Effect of green tea supplementation on insulin sensitivity in Sprague-Dawley rats J. Agric. Food Chem 2004 52 643 648 14759162 10.1021/jf030365d

[182] Bain JR Stevens RD Wenner BR Ilkayeva O Muoio DM Newgard CB Metabolomics applied to diabetes research: Moving from information to knowledge Diabetes 2009 58 2429 2443 19875619 10.2337/db09-0580 PMC2768174

[183] Iwai K Kim MY Onodera A Matsue H Alpha-glucosidase inhibitory and antihyperglycemic effects of polyphenols in the fruit of Viburnum dilatatum Thunb J. Agric. Food Chem 2006 54 4588 4592 16787002 10.1021/jf0606353

[184] Tadera K Minami Y Takamatsu K Matsuoka T Inhibition of alpha-glucosidase and alpha-amylase by flavonoids J. Nutr. Sci. Vitaminol. (Tokyo) 2006 52 149 153 16802696 10.3177/jnsv.52.149

[185] Lo Piparo E Scheib H Frei N Williamson G Grigorov M Chou CJ Flavonoids for controlling starch digestion: Structural requirements for inhibiting human alpha-amylase J. Med. Chem 2008 51 3555 3561 18507367 10.1021/jm800115x

[186] Kim JS Kwon CS Son KH Inhibition of alpha-glucosidase and amylase by luteolin, a flavonoid Biosci. Biotechnol. Biochem 2000 64 2458 2461 11193416 10.1271/bbb.64.2458

[187] Funke I Melzig MF Effect of different phenolic compounds on alpha-amylase activity: Screening by microplate-reader based kinetic assay Pharmazie 2005 60 796 797 16259133

[188] Narita Y Inouye K Kinetic analysis and mechanism on the inhibition of chlorogenic acid and its components against porcine pancreas alpha-amylase isozymes I and II J. Agric. Food Chem 2009 57 9218 9225 19807164 10.1021/jf9017383

[189] McDougall GJ Shpiro F Dobson P Smith P Blake A Stewart D Different polyphenolic components of soft fruits inhibit alpha-amylase and alpha-glucosidase J. Agric. Food Chem 2005 53 2760 2766 15796622 10.1021/jf0489926

[190] Lee YA Cho EJ Tanaka T Yokozawa T Inhibitory activities of proanthocyanidins from persimmon against oxidative stress and digestive enzymes related to diabetes J. Nutr. Sci. Vitaminol. (Tokyo) 2007 53 287 292 17874835 10.3177/jnsv.53.287

[191] Adisakwattana S Charoenlertkul P Yibchok-Anun S alpha-Glucosidase inhibitory activity of cyanidin-3-galactoside and synergistic effect with acarbose J. Enzyme Inhib. Med. Chem 2009 24 65 69 18615280 10.1080/14756360801906947

[192] Adisakwattana S Ngamrojanavanich N Kalampakorn K Tiravanit W Roengsumran S Yibchok-Anun S Inhibitory activity of cyanidin-3-rutinoside on alpha-glucosidase J. Enzyme Inhib. Med. Chem 2004 19 313 316 15558946 10.1080/14756360409162443

[193] Matsui T Ueda T Oki T Sugita K Terahara N Matsumoto K alpha-Glucosidase inhibitory action of natural acylated anthocyanins. 2. alpha-Glucosidase inhibition by isolated acylated anthocyanins J. Agric. Food Chem 2001 49 1952 1956 11308352 10.1021/jf0012502

[194] Matsui T Ueda T Oki T Sugita K Terahara N Matsumoto K alpha-Glucosidase inhibitory action of natural acylated anthocyanins. 1. Survey of natural pigments with potent inhibitory activity J. Agric. Food Chem 2001 49 1948 1951 11308351 10.1021/jf001251u

[195] Welsch CA Lachance PA Wasserman BP Effects of native and oxidized phenolic compounds on sucrase activity in rat brush border membrane vesicles J. Nutr 1989 119 1737 1740 2600679 10.1093/jn/119.11.1737

[196] Hanamura T Hagiwara T Kawagishi H Structural and functional characterization of polyphenols isolated from acerola (Malpighia emarginata DC.) fruit Biosci. Biotechnol. Biochem 2005 69 280 286 15725651 10.1271/bbb.69.280

[197] Lee DS Lee SH Genistein, a soy isoflavone, is a potent alpha-glucosidase inhibitor FEBS Lett 2001 501 84 86 11457461 10.1016/s0014-5793(01)02631-x

[198] Adisakwattana S Chantarasinlapin P Thammarat H Yibchok-Anun S A series of cinnamic acid derivatives and their inhibitory activity on intestinal alpha-glucosidase J. Enzyme Inhib. Med. Chem 2009 24 1194 1200 19772492 10.1080/14756360902779326

[199] Chauhan A Gupta S Mahmood A Effect of tannic acid on brush border disaccharidases in mammalian intestine Indian J. Exp. Biol 2007 45 353 358 17477307

[200] Schafer A Hogger P Oligomeric procyanidins of French maritime pine bark extract (Pycnogenol) effectively inhibit alpha-glucosidase Diabetes Res. Clin. Pract 2007 77 41 46 17098323 10.1016/j.diabres.2006.10.011

[201] Ohno T Kato N Ishii C Shimizu M Ito Y Tomono S Kawazu S Genistein augments cyclic adenosine 3′5′-monophosphate(cAMP) accumulation and insulin release in MIN6 cells Endocr. Res 1993 19 273 285 7508378 10.1080/07435809309026682

[202] Liu D Zhen W Yang Z Carter JD Si H Reynolds KA Genistein acutely stimulates insulin secretion in pancreatic beta-cells through a cAMP-dependent protein kinase pathway Diabetes 2006 55 1043 1050 16567527 10.2337/diabetes.55.04.06.db05-1089

[203] Jonas JC Plant TD Gilon P Detimary P Nenquin M Henquin JC Multiple effects and stimulation of insulin secretion by the tyrosine kinase inhibitor genistein in normal mouse islets Br. J. Pharmacol 1995 114 872 880 7773549 10.1111/j.1476-5381.1995.tb13285.x PMC1510214

[204] Sorenson RL Brelje TC Roth C Effect of tyrosine kinase inhibitors on islets of Langerhans: Evidence for tyrosine kinases in the regulation of insulin secretion Endocrinology 1994 134 1975 1978 8137766 10.1210/endo.134.4.8137766

[205] Hsu FL Liu IM Kuo DH Chen WC Su HC Cheng JT Antihyperglycemic effect of puerarin in streptozotocin-induced diabetic rats J. Nat. Prod 2003 66 788 792 12828463 10.1021/np0203887

[206] Li Y Kim J Li J Liu F Liu X Himmeldirk K Ren Y Wagner TE Chen X Natural anti-diabetic compound 1,2,3,4,6-penta-O-galloyl-D-glucopyranose binds to insulin receptor and activates insulin-mediated glucose transport signaling pathway Biochem. Biophys. Res. Commun 2005 336 430 437 16137651 10.1016/j.bbrc.2005.08.103

[207] Pinto Mda S Kwon YI Apostolidis E Lajolo FM Genovese MI Shetty K Potential of Ginkgo biloba L. leaves in the management of hyperglycemia and hypertension using *in vitro* models Bioresour. Technol 2009 100 6599 6609 19665890 10.1016/j.biortech.2009.07.021

[208] Kashket S Paolino VJ Inhibition of salivary amylase by water-soluble extracts of tea Arch. Oral Biol 1988 33 845 846 2476976 10.1016/0003-9969(88)90110-0

[209] Koh LW Wong LL Loo YY Kasapis S Huang D Evaluation of different teas against starch digestibility by mammalian glycosidases J. Agric. Food Chem 2009 58 148 154 20050703 10.1021/jf903011g

[210] Kusano R Andou H Fujieda M Tanaka T Matsuo Y Kouno I Polymer-like polyphenols of black tea and their lipase and amylase inhibitory activities Chem. Pharm. Bull. (Tokyo) 2008 56 266 272 18310934 10.1248/cpb.56.266

[211] Kwon YI Apostolidis E Kim YC Shetty K Health benefits of traditional corn, beans, and pumpkin: *In vitro* studies for hyperglycemia and hypertension management J. Med. Food 2007 10 266 275 17651062 10.1089/jmf.2006.234

[212] Kwon YI Apostolidis E Shetty K *In vitro* studies of eggplant (Solanum melongena) phenolics as inhibitors of key enzymes relevant for type 2 diabetes and hypertension Bioresour. Technol 2008 99 2981 2988 17706416 10.1016/j.biortech.2007.06.035

[213] da Silva Pinto M Kwon YI Apostolidis E Lajolo FM Genovese MI Shetty K Functionality of bioactive compounds in Brazilian strawberry (Fragaria x ananassa Duch.) cultivars: Evaluation of hyperglycemia and hypertension potential using *in vitro* models J. Agric. Food Chem 2008 56 4386 4392 18522404 10.1021/jf0732758

[214] Yao Y Sang W Zhou M Ren G Antioxidant and α-Glucosidase inhibitory activity of colored grains in china J. Agric. Food Chem 2009 58 770 774 19904935 10.1021/jf903234c

[215] Kwon Y Apostolidis E Shetty K Inhibitory potential of wine and tea against alpha-amylase and alpha-glucosidase for management of hyperglycemia linked to type 2 diabetes J. Food Biochem 2008 32 15 31

[216] Nordentoft I Jeppesen PB Hong J Abudula R Hermansen K Increased insulin sensitivity and changes in the expression profile of key insulin regulatory genes and beta cell transcription factors in diabetic KKAy-mice after feeding with a soy bean protein rich diet high in isoflavone content J. Agric. Food Chem 2008 56 4377 4385 18522411 10.1021/jf800504r

[217] Roffey B Atwal A Kubow S Cinnamon water extracts increase glucose uptake but inhibit adiponectin secretion in 3T3-L1 adipose cells Mol. Nutr. Food Res 2006 50 739 745 16835867 10.1002/mnfr.200500253

[218] Khan SA Priyamvada S Arivarasu NA Khan S Yusufi AN Influence of green tea on enzymes of carbohydrate metabolism, antioxidant defense, and plasma membrane in rat tissues Nutrition 2007 23 687 695 17679048 10.1016/j.nut.2007.06.007

[219] Govorko D Logendra S Wang Y Esposito D Komarnytsky S Ribnicky D Poulev A Wang Z Cefalu WT Raskin I Polyphenolic compounds from Artemisia dracunculus L. inhibit PEPCK gene expression and gluconeogenesis in an H4IIE hepatoma cell line Am. J. Physiol. Endocrinol. Metab 2007 293 E1503 E1510 17848630 10.1152/ajpendo.00420.2007

